# Gold Nanoparticle Adsorption and Uptake are Directed by Particle Capping Agent

**DOI:** 10.1002/smsc.202500060

**Published:** 2025-05-19

**Authors:** Rashad Kariuki, Rowan Penman, Alexander D. Newbold, Kalpani A. Mirihana, Pierre H. A. Vaillant, Tilly P. Shepherd, Nastaran Meftahi, Gary Bryant, Kislon Voïtchovsky, Claudia Contini, Andrew Hung, Kevion K. Darmawan, Charlotte E. Conn, Saffron J. Bryant, Andrew J. Christofferson, Aaron Elbourne

**Affiliations:** ^1^ School of Science STEM College RMIT University Melbourne Victoria 3001 Australia; ^2^ Physics Department Durham University Durham DH1 3LE UK; ^3^ Department of Civil and Construction Engineering Swinburne University of Technology Melbourne Victoria 3122 Australia; ^4^ Department of Life Sciences Imperial College London London SW7 2AZ UK

**Keywords:** adsorption, atomic force microscopy, gold nanoparticles, ligands, membrane interactions, molecular dynamics simulations, supported lipid bilayers

## Abstract

Nanomaterials are revolutionizing the development of novel therapies, with applications ranging from drug delivery and diagnostics to controlling specific biological processes. However, the specific interactions that govern nanomaterial behavior in biological systems remain difficult to elucidate due to the complex dynamic nature of the lipid bilayer environment. Here, a combination of atomic force microscopy and molecular dynamics simulations is used to discover the precise mechanisms by which various ligand‐capped 5 nm gold nanoparticles (AuNPs) interact with supported lipid bilayers of pure fluid phospholipids (1,2‐di(9Z‐octadecenoyl)‐sn‐glycero‐3‐phosphocholine (DOPC)). When the ligand capping agent is altered, differences in adsorption and bilayer disruption as a function of capping agent size and charge are observed. Weakly physiosorbed ligands enable the absorption of the AuNP into the bilayer's hydrophobic core, whereas more strongly adsorbed ligands inhibit the complete insertion of the AuNP. However, ligand‐dependent headgroup interactions can lead to interfacial adhesion or inhibition of adsorption. These results reveal that the interaction of AuNPs with biological membranes varies depending on the specific capping agent. Notably, the mechanisms may involve cooperative (or synergistic) effects with membrane components, highlighting the importance of understanding these interactions at molecular resolution.

## Graphical abstract

This study explores how ligand‐coated gold nanoparticles (NPs) interact with model membranes. Combining atomic force microscopy (AFM) and molecular dynamics (MD) simulations, it shows how coating properties affect adsorption. The AFM‐MD approach offers a predictive platform for nanoparticle design and toxicity, enabling tailored nanomedicine development and forecasting of NP‐induced cytotoxic effects.

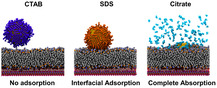

## 1 Introduction

Nanomaterials have distinctive physical,^[^ ^1^, ^2^ ^]^ optical,^[^ ^3^, ^4^ ^]^ and self‐assembly^[^ ^5^, ^6^ ^]^ properties that give rise to unique nanoscale behavior, allowing for alternative therapeutic,^[^ ^7^, ^8^ ^]^ diagnostic,^[^ ^9^, ^10^ ^]^ or pharmaceutical vector capabilities.^[^ ^11^, ^12^ ^]^ The core feature of therapeutic nanomaterials is that carry out their desired cellular function, they must inevitably interact,^[^ ^13^, ^14^ ^]^ bypass,^[^ ^15^, ^16^ ^]^ or shield^[^ ^17^, ^18^ ^]^ themselves from the cell membrane, which is composed of self‐assembled phospholipids.^[^ ^19^ ^]^ There is a significant amount of research focused on tailoring nanoparticle (NP) composition via synthesis techniques.^[^ ^20^, ^21^ ^]^ Specific types of cellular interactions are promoted/hindered through control of NP morphology,^[^ ^22^, ^23^ ^]^ composition,^[^ ^24^, ^25^ ^]^ and surface chemistry,^[^ ^26^, ^27^ ^]^ with the ideal end goal of complete control of NP behavior in vivo via selection of NP properties. However, this task has proven to be extremely difficult due to the complexities of the cell, as it hosts a wide variety of dynamically regulated biomolecules,^[^ ^28^, ^29^ ^]^ altering subsequent NP–membrane interactions^[^ ^30^, ^31^ ^]^ and complicating specific molecular characterization.^[^ ^32^, ^33^ ^]^ Model membranes offer a platform to overcome these complexities via simplified composition, such as pure phospholipid bilayers, which can be scaled up with biomolecules into various morphologies to represent a range of cellular types/environments.^[^ ^34^, ^35^ ^]^ This allows for a systematic investigation of contributing effects to the interaction process. SLBs are routinely used to study biophysical processes of membranes as they offer precise control of their composition, environment, and structural properties. SLBs can also represent a range of cellular environments.^[^ ^36^ ^]^

Amongst a wide range of nanomaterials being investigated, gold nanoparticles (AuNPs) have gained significant biomedical clinical interest due to their biocompatibility,^[^ ^37^, ^38^, ^39^, ^40^, ^41^ ^]^ ease of synthesis,^[^ ^42^, ^43^, ^44^, ^45^ ^]^ and alterable surface chemistry.^[^ ^46^, ^47^, ^48^, ^49^, ^50^ ^]^ Their biocompatibility and properties have been routinely demonstrated in cell culture,^[^ ^51^, ^52^, ^53^ ^]^ in vivo animal models,^[^ ^54^, ^55^, ^56^, ^57^ ^]^ and various clinical trials.^[^ ^58^, ^59^, ^60^ ^]^ NP adsorption to lipid membranes is largely influenced by the free energy landscape of the system, determined by the sum of molecular forces between the NP, the membrane, and the solvation environment (i.e., van der Waals (vdW), Coulombic, hydrogen bonding, etc.).^[^ ^61^, ^62^ ^]^ The specific free energy landscape of an NP changes as a function of its surface chemistry,^[^ ^63^, ^64^, ^65^ ^]^ which may be altered via the addition of ligands onto its surface,^[^ ^66^, ^67^ ^]^ thereby changing its interactions with biological systems such as lipid membranes.^[^ ^68^, ^69^, ^70^, ^71^, ^72^, ^73^ ^]^ Control of NP adsorption via the addition of ligands is a significant area of biomedical research, as ligand properties can be tailored to target cellular environments,^[^ ^70^, ^71^, ^72^ ^]^ deliver therapies,^[^ ^69^, ^74^, ^75^ ^]^ and control biological interactions.^[^ ^17^, ^73^, ^76^ ^]^ Additionally, ligands are often necessary for the synthesis of various NPs, as they act as reducing agents, nucleating regulators, or aggregation‐stabilizers which may remain on the surface postsynthesis.^[^ ^66^, ^77^, ^78^ ^]^ Furthermore, in vivo surface adsorbed molecules (i.e., biological coronas) may act as additional unintentional pseudo‐ligands (or surface adsorbed molecules).^[^ ^79^, ^80^, ^81^, ^82^, ^83^, ^84^, ^85^, ^86^ ^]^ Therefore, understanding the influence of ligand properties on the interaction process is crucially important for the development of biomedical therapies.

During biomembrane interactions, NPs may adsorb to the bilayer interface, embed within the bilayer core, or fail to interact with the bilayer. The bilayer interface is primarily hydrophilic and the core hydrophobic, allowing for selection based on ligand hydrophobicity.^[^ ^69^, ^72^, ^87^ ^]^ NP interactions are influenced by the relationship between the NP ligand and the membrane composition. As cell bilayers are chemically heterogeneous, investigations into fundamental interactions are complicated by the variety of cellular compositions.^[^ ^88^ ^]^ Similarly, NP characterization is also complicated by various NP properties and the limitations of analytical instrumentation only look for very specific properties.^[^ ^89^ ^]^ The complexities of analysis of NP‐membrane systems and their environments are compounded during investigations of fundamental NP–membrane interactions. Additionally, the extensive material and mechanical descriptors for NP materials and membranes are quite vast, which further convolutes data collection.^[^ ^90^ ^]^ Due to these factors, fundamental information about the effects of ligands on NP adsorption in a controlled setting remain limited. It should also be highlighted that a majority of clinically relevant NP‐cellular studies have an increased focus on holistic outcomes, i.e., cell death,^[^ ^91^ ^]^ organ dysfunction,^[^ ^92^ ^]^ or metabolic alterations^[^ ^52^ ^]^ rather than fundamental molecular interactions.^[^ ^93^, ^94^, ^95^, ^96^ ^]^ Instead, larger‐scale properties are often favored, such as membrane estructure,^[^ ^97^ ^]^ fluidity changes,^[^ ^98^, ^99^ ^]^ or cell lysis.^[^ ^100^ ^]^

The complexity of the cellular environment limits the accuracy of analytical techniques, as currently there are few systematic methods for tracking interactions between single NPs and biomembrane at molecular resolution. To fully understand the AuNP adsorption process, the structure–function relationship in AuNP‐ligand‐membrane systems and subsequent adsorption behavior must be established. Achieving a fundamental molecular understanding of NP–membrane interactions will lead to more advanced and safer clinical therapies, enabled by rational design, allowing higher predictability of outcomes.^[^ ^90^, ^101^, ^102^ ^]^ Here, we present key insights into the adsorption mechanisms between supported lipid bilayers (SLBs) and 5 nm AuNPs with varying capping agents (or ligands), using a simplified system allowing clear interpretation of the results. To do this, we investigate the interaction of ligand‐capped AuNPs with a fluid phase phospholipid bilayer, using a combination of atomic force microscopy (AFM) and molecular dynamics (MD) simulations.

**Figure 1:**
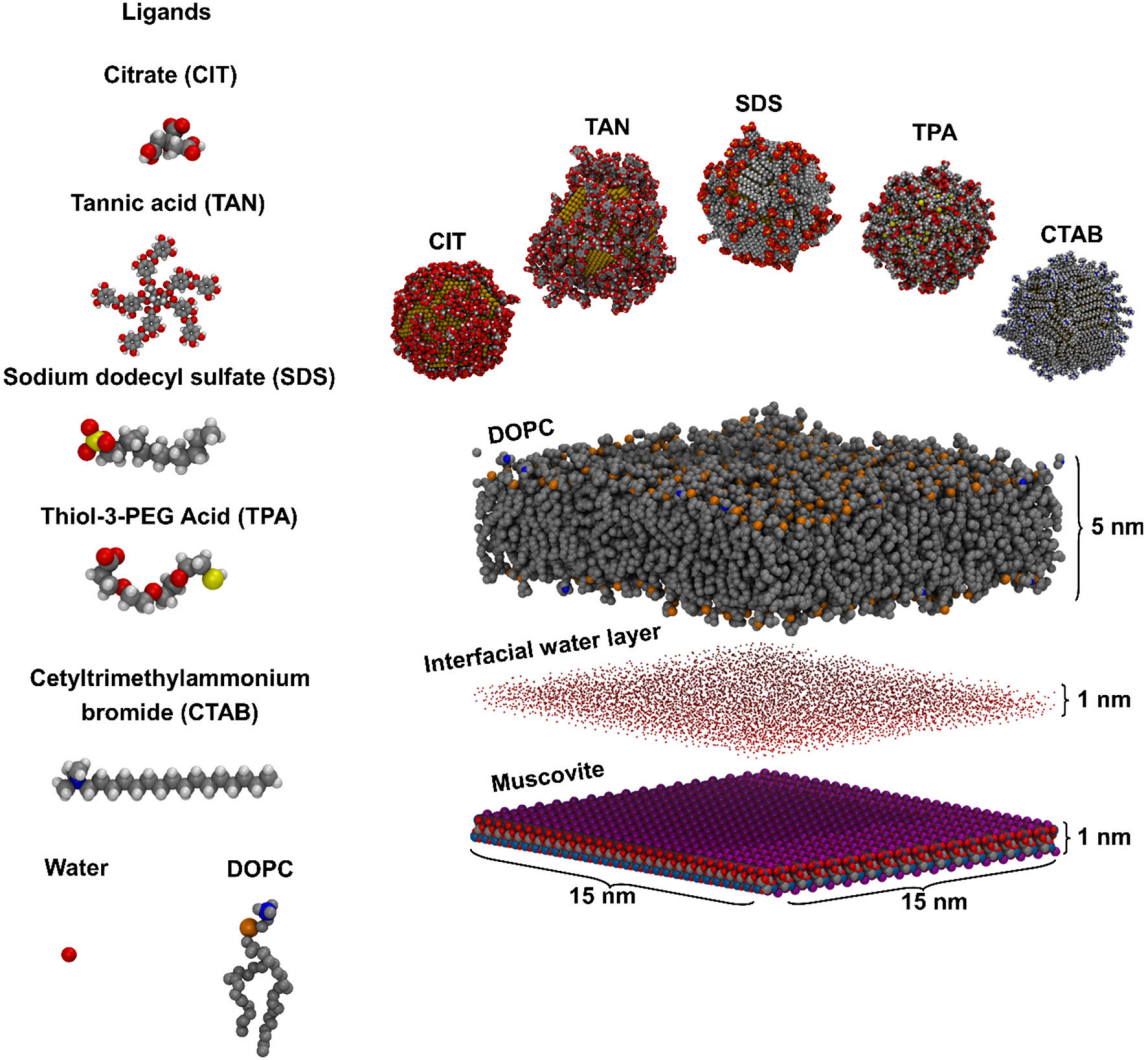
MD simulation setup for the investigation of the effects of ligand capping and SLB composition on AuNP adsorption.

Noble metal NPs (such as AuNPs) are commonly functionalized with a self‐assembled monlayer surface coating, depending on the application. Here, the ligands were selected for their biomedical relevance and differences in key structural features such as carbon chain length and functional group. Specifically, we investigated AuNPs capped by citrate (CIT), tannic acid (TAN), sodium dodecyl sulphate (SDS), thiol‐3‐polyethylene glycol acid (TPA), and cetyltrimethylammonium bromide (CTAB) to establish a structure–function relationship for NP–membrane interactions. The model biomembrane was kept consistent, as a fluid phase (Lα), pure 1,2‐di(9Z‐octadecenoyl)‐sn‐glycero‐3‐phosphocholine (DOPC), self‐assembled atop a muscovite (mica) surface (**Figure** **1**). A zwitterionic phospholipid was chosen to avoid probing any obvious electrostatic interactions originating from the bilayer. Figure 1 shows a stylized schematic of these systems using the MD simulation components.

Overall, these results agree with existing literature on the generalized mechanisms of ligand dissociation and the effects of NP adsorption onto bilayers.^[^ ^97^, ^103^, ^104^, ^105^, ^106^, ^107^ ^]^ In addition, this study establishes a framework for the adsorption of 5 nm AuNPs to SLBs and developed an atomistic model to explain the observed differences in adsorption. The adsorption differences between hard and soft NPs have been explored.^[^ ^108^ ^]^ Soft NPs can more easily undergo conformational changes,^[^ ^30^, ^109^ ^]^ which facilitates incorporation into biomembranes, whilst harder NPs are rigid, and hence, more resistant to direct adsorption into a biomembrane.^[^ ^104^, ^105^, ^107^, ^110^, ^111^, ^112^, ^113^ ^]^ Additionally, the effects of ligand morphology on NP adsorption behavior have shown that larger ligand shapes^[^ ^114^ ^]^ and higher ligand densities increase the cost of adsorption.^[^ ^6^ ^]^ However, the mechanism for biomembrane uptake of hybrid hard–soft NP systems, such as the ligand‐capped metallic NPs studied in this work, remains largely unexplored. The ligand capping agent is known to influence the initial interaction between the AuNPs and the bilayers. By contrast, where the ligand density sheilds the NP, or the ligand affinty does not promote dissociation of the cap, core composition may have negligible influence on NP adsorption. This platform enables the effects of the capping agent to be differentiated from the effects of the NP core, whilst also characterizing the distinct molecular mechanisms by which various hybrid hard–soft (i.e., ligand‐coated) NPs interact with SLBs.

The various NP behaviors observed, namely interfacial adsorption, absorption, or inhibition of adsorption, have biomedical implications. The ability of NPs to localize within the membrane has implications for drug delivery^[^ ^115^ ^]^ while adherence to the interface has implications in cellular tagging^[^ ^116^ ^]^ and hindering adsorption enables stealth (immune cell evasion) properties.^[^ ^17^ ^]^ Controlling the localization fate of an NP is a primary objective of pharmacokinetics, as control over specific biological pathways may reduce toxicity.^[^ ^117^ ^]^ The adsorption of various AuNPs to different solution‐based phospholipid bilayers, such as liposomal/vesicle models, has been previously investigated.^[^ ^103^, ^118^ ^]^ Often, these studies use high‐resolution optical or electron microscopy methods, as well as scattering or spectroscopic techniques to investigate adsorption mechanisms. Overall, these studies report the general rearrangement and disruption of lipids as a function of NP adsorption, which are rationalized in the context of biophysical models. These techniques, however, often do not study the precise molecular NP‐bilayer adsorption of single NPs due to the increased difficulty of imaging NP interactions at this size regime using classical experimental techniques. Currently, contradictory results have been observed for the influence of ligand parameters on uptake. For example, Jinag et al. showed an increased uptake of cationic 2,4,6 nm AuNPs in HeLa cells,^[^ ^119^ ^]^ whilst Canepa et al.'s MD coarse‐grained models showed minimal electrostatic adsorption bias effects from either cationic or anionic 2 nm AuNPs to zwitterionic membranes.^[^ ^120^ ^]^ These discrepancies in adsorption behavior can be attributed to the multifaceted nature of adsorption, where ligand charge, solvent interactions, and ligand affinities interplay and give rise to dynamic NP‐adsorption behavior, emphasizing the need for more simplified systematic studies on the role of NP‐ligand properties. There have been various investigations into the role of ligand charge,^[^ ^72^, ^121^ ^]^ ligand morphology and composition,^[^ ^96^ ^]^ ligand hydrophobicity,^[^ ^70^ ^]^ and ligand NP‐affinity^[^ ^72^, ^104^ ^]^ on NP adsorption. In parallel, SLB models to elucidate and compare the effects of various physiosorbed and chemisorbed NPs have been developed at the coarse‐grain level,^[^ ^106^ ^]^ as well as other experimental and coarse‐grained MD floating bilayer studies.^[^ ^110^ ^]^ However, currently, there are no fully atomistic models that also attempt to compare NP‐ligand properties and their role in NP adsorption in comparison with experimental SLB models. Here we show that ligand adsorption is not dependent on a singular ligand property, but the interplay of various ligand properties and their interaction with the lipids, the solvent environment, and the affinity of the NP core.

In our study, we utilize both AFM and MD to investigate high and low spatiotemporal ranges, which allows large‐scale diffusive behavior (AFM) to be linked to molecular behavior (MD), providing strong insights into the overall behavior of the NP‐SLB system. Although this simplified homogenous DOPC platform cannot fully replicate the dynamic processes and composition of natural bilayers, this work establishes an adaptable protocol for determining the effects of the AuNP ligand coating on the SLB adsorption process, which can be further tailored toward numerous NP types, ligands, and relevant biomimetic membrane compositions. Advancing NP‐bilayer compositional complexity allows for a more accurate representation of potential in vivo NP‐bio phenomena and will enable the systematic analysis of the effect of additional/alternative NP‐SLB properties on NP adsorption. Moreover, this platform provides a fundamental tool for screening adsorption capabilities of various NP‐bilayer systems, thereby advancing the preclinical development of these therapies.

## 2 Results

### 2.1 AFM

**Figure 2:**
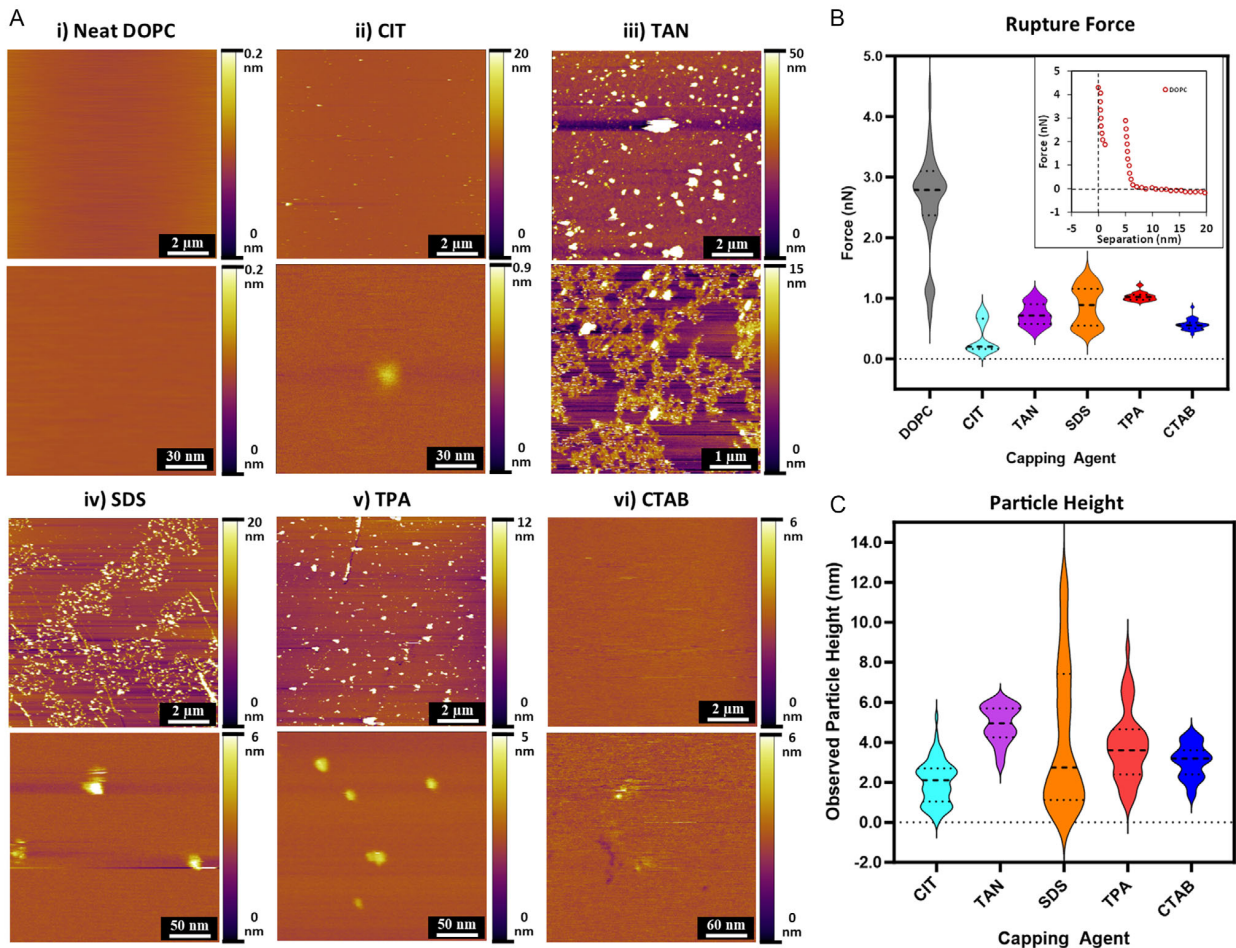
A) AFM topographic images of i) Neat DOPC, ii) CIT, iii) TAN, iv) SDS, v) TPA, and (vi) CTAB, with low (top) and high (bottom) magnifications shown for each case. B) AFM force spectroscopy graph of rupture forces of NP‐membrane systems (2A i–vi). C) Particle heights of NP‐membrane systems (2A ii–vi).

High‐resolution amplitude‐modulated‐AFM imaging was conducted in liquid using small‐amplitude imaging protocols previously developed for interfacial imaging of soft matter interfaces,^[^ ^122^, ^123^ ^]^ viscous fluids,^[^ ^124^ ^]^ and ionic solutes.^[^ ^125^ ^]^ For all experiments, surface‐adsorbed flat lipid bilayers were formed via the previously reported vesicle fusion method.^[^ ^126^, ^127^ ^]^ **Figure** **2**A shows representative low‐resolution (10 × 10 μm) and high‐resolution (150 × 150 nm) AFM height images for SLBs of (i) neat DOPC and (ii–vi) the different ligands. For neat DOPC, the bilayer surface is relatively homogeneous and commensurate with a flat, continuous adsorbed lipid bilayer. In a standard AFM experiment, such as that performed here, no direct chemical data are provided, meaning that confirming the presence of a continuous lipid bilayer cannot be achieved by images alone since the bilayer appears smooth, akin to the underlying mica surface. To confirm the presence of the lipid bilayer, we used force spectroscopy (Figure 2B); the AFM tip is moved toward the substrate at a selected location while tracking the force it experiences. These apparent force curves show a characteristic step as the tip ruptures the bilayer (Figure 2B, inset) at a distance of ≈5 nm, when pressing with more than ≈2.78 ± 0.96 nN force. The rupture force depends on many parameters, including the tip geometry, lipid composition, and the environment, but it unambiguously indicates the presence of a bilayer. Past the rupture point, the cantilever then presses on the mica substrate supporting the DOPC membrane, confirming a total bilayer thickness close to ≈5 nm, consistent with previous AFM investigations.^[^ ^128^ ^]^ The imaging force for these experiments is roughly ≈0.2 nN (see Figure S1, Supporting Information), which is below the threshold force for movement of individual proteins or lipids,^[^ ^129^, ^130^, ^131^ ^]^ as such, it is assumed to have negligible disturbance on system dynamics.

Following confirmed SLB formation, a 15 μL aliquot of the AuNP solution (5 × 10^13^ particles mL^−1)^ was introduced to the system, meaning that ≈5 × 10^10^ particles could equilibrate with the SLB. The AuNPs were equilibrated with the surface for ≈30 min before imaging. Average rupture forces for the SLBs can be seen in **Table** **1**. A different location is randomly selected for each set of measurements to gather a representative sample of the bilayer integrity across the system. This is necessary because we cannot identify where single AuNPs are at any given moment. The underlying mica surface is circular with a diameter of 1 cm, equivalent to ≈700 particles μm^−2^. It should be noted, however, that this is only an estimate, and the number of surface‐bound AuNPs is expected to be lower than this, as not all particles will migrate to the interface from solution immediately. Additionally, there may be some ligand‐dependent aggregation, as shown by complementary dynamic light scattering (DLS) measurements (see Figure S2, Supporting Information) which may influence their dispersion and membrane settling rate. Transmission electron microscopy (TEM) images of the ligand‐capped 5 nm AuNPs can be found in Figure S3, Supporting Information, further highlighting their individual particle size, monodispersity, and aggregation behavior. Cluster analysis (see Figure S4, Supporting Information) was performed on AFM topography images using Gwyddion scanning probe microscopy (SPM) software to distinguish between individual NPs and NP clusters adsorbed onto the SLB. The analysis was based on a height threshold approach, where features exceeding a defined height relative to the bilayer surface were segmented and identified. This threshold was selected to differentiate between single NPs and aggregates, allowing for statistical characterization of particle populations. The resulting data provided insights into the distribution, size, and clustering behavior of NPs interacting with the lipid membrane. Additional AFM phase data can further be found in Figure S5, Supporting Information, this does not show any noticeable difference from AFM height data.

**Table 1:** Rupture forces and height profile summary.

System	DOPC	CIT	TAN	SDS	TPA	CTABb)
Rupture forcea) [*n* = 50]	2.78 ± 0.96 nN	0.35 ± 0.25 nN	0.73 ± 0.19 nN	0.88 ± 0.33 nN	1.03 ± 0.07 nN	0.57 ± 0.09 nN
Particle height [*n* = 50]	N/A	2.00 ± 1.07 nm	4.91 ± 0.90 nm	4.20 ± 3.50 nm	3.86 ± 1.74 nm	3.06 ± 0.82 nm

a) See Figure S6, Supporting Information for rupture force evolution profile;

b) CTAB (*n* = 5, as particles were observed infrequently).

AFM imaging and force spectroscopy revealed that the various capped AuNPs all lowered bilayer integrity during SLB interaction. The degree of SLB‐AuNP penetration/adsorption was significantly influenced by the composition of the ligand coating, where smaller ligands such as CIT showed greater adsorption depths as compared to larger ligands such as TAN, SDS, and TPA which showed primarily interfacial activity. CTAB showed only occasional bilayer adsorption, and the SLB was generally devoid of surface features. This is in contrast to the other systems (CIT, TAN, SDS, and TPA) where surface coverage was high. While the degree of bilayer penetration was linked to higher rates of membrane disruption (e.g., CIT showed the highest change in rupture force and membrane penetration), systems such as TAN, SDS, TPA, and CTAB which showed primarily interfacial interaction, also exhibited disruptive lipid organization mechanisms despite the lack of complete adsorption into the SLB core. This suggests that disruptive membrane mechanisms can exist via interfacial interaction without the need for full SLB absorption.

Figure 2A(i–vi) shows the equilibrated DOPC‐SLB‐AuNP‐ligand systems, the CIT system surface still appears relatively smooth with randomly isolated AuNP protrusions. By contrast, TAN and TPA show an abundance of large‐scale AuNP surface aggregates. SDS also shows a large abundance of large‐scale surface aggregates, however, these clusters appear to be joined by longer interconnected “webs”, which may be attributed to the attempted micellization of dissociated SDS molecules as they are not covalently bound to the AuNP surface and may also behave as a linker between AuNP clusters.^[^ ^132^ ^]^ The clustering of the AuNPs is unsurprising given the aggregation seen in the DLS data (Figure S2, Supporting Information), with aggregate peaks of order ≈100 nm for all samples. Similar to TAN, SDS, and TPA systems, CTAB shows some bilayer protrusions on closer inspection, however, it lacks the surface aggregates seen in the other systems.

Different AuNP ligand compositions resulted in different SLB interactions, as can be seen visually in the AFM results. From a biomechanical perspective, AFM force spectroscopy revealed that the addition of the AuNPs to the SLB system led to membrane disruption effects and lowered the bilayer integrity of each system. The AFM tip measures the localized bilayer rupture force (bilayer integrity) next to the site of NP adsorption. Figure 2B shows the drop in rupture force of the various SLBs, and the CIT system shows the largest decrease in rupture force to 0.35 ± 0.25 nN. This is then followed by CTAB at 0.57 ± 0.09 nN, TAN at 0.73 ± 0.19 nN, SDS at 0.88 ± 0.33 nN, and TPA at 1.03 ± 0.07 nN. Individual representative force curves are shown in Figure S7, Supporting Information. Interestingly, both the CIT and the CTAB had the highest change in rupture force but also had the fewest visible surface aggregates, which may indicate higher adsorption rates into the membrane (as the AuNPs are not as visible on the surface but still able to induce mechanical changes). AFM imaging was further able to elucidate the overall membrane penetration depth of the AuNP protrusions with the SLBs. Figure 2C shows the average bilayer particle height profiles, with lower height measurements indicative of high penetration depths into the bilayer. The CIT‐coated AuNPs had the lowest height of 2.00 ± 1.07 nm, which is lower than the apparent normal AuNP height of ≈5 nm, suggesting successful membrane absorption. The CTAB‐coated AuNPs had a recorded height of 3.06 ± 0.82 nm, indicative of interfacial adsorption (or occasional adsorption), followed by TPA at 3.86 ± 1.74 nm, SDS at 4.20 ± 3.50 nm, and TAN at 4.91 ± 0.90 nm. For the CIT, the apparent lowered height of the bilayer protrusions, along with a large change in the rupture force, is also indicative of NP adsorption within the bilayer. The larger average height profiles for TAN, SDS, TPA, and CTAB suggest interfacial adsorption, however, the measured change in rupture forces suggests a mechanism of bilayer destabilization that is exclusive of membrane penetration. It should be further noted for CIT, SDS, TPA, and CTAB that while the bilayer integrity was disrupted on a molecular level, the presence of the relatively smooth SLB surface indicates the ability of the AuNP to incorporate or interact with the bilayer without significant macroscale changes to the bilayer structure, in contrast to TAN where the formation of lipid domains can be observed.

Previous work on the interactions of coated AuNPs with bilayers has shown that citrate‐coated AuNPs undergo adsorption to phosphocholine bilayer interfaces, as well as cause changes to lipid organization and affect bilayer fluidity.^[^ ^97^, ^103^, ^104^, ^105^, ^107^ ^]^ The mechanism of adsorption of citrated‐capped AuNPs to bilayers has been suggested to involve the dissociation of the citrate ligand coating of the AuNP surface and subsequent NP adsorption due to the hydrophobic contacts between the AuNP surface and the lipid tails.^[^ ^103^, ^104^, ^105^, ^107^ ^]^ This allows the AuNP to incorporate itself into the membrane and alter the membrane ordering without significant macroscale alteration. It should be highlighted that while an overall disruptive mechanism is observed here, in curved membranes such as liposomes, the opposite effect (ordering) can also be observed, that is, AuNPs causing a fluid‐gel‐like transition during adsorption.^[^ ^118^ ^]^ The differences in NP adsorption between the two systems may be rationalized by geometric constraints on lipid packing due to the morphology, leading to alternative membrane behavior.

In the case of TAN, the development of conserved lipid domains, as opposed to the smooth planar bilayer observed with the neat DOPC or during the adsorption of the other AuNPs, suggests significant membrane disruption during interaction that does not occur in the other AuNP systems. Additionally, the height profiles suggest this disruption mechanism is exclusive from membrane penetration, in contrast to CIT, where the membrane destabilization coincided with adsorption. This disruptive bilayer effect may be due to the abundance of AuNP aggregates inducing a mechanical destabilization effect. Additionally, it has been observed that tannic acid molecules can induce the formation of reactive oxygen species, which can affect membrane integrity,^[^ ^133^ ^]^ and this may happen during membrane interaction with the AuNP complex, as well as from additional TAN molecules that may be only partially associated with the NP‐complex.

Specific reports of SDS‐coated AuNPs in lipid bilayer adsorption appear to be lacking in the literature. This may be attributed to the widespread use of SDS as a detergent, as it can solubilize bilayers,^[^ ^134^ ^]^ which may be disruptive for the analysis of adsorption data, making it less suitable for certain biomedical studies. As a result, researchers may seek alternative coatings that provide more controlled interactions for therapeutic applications. However, SDS still has a role as a potential surfactant in the synthesis of relevant biomedical NPs,^[^ ^135^ ^]^ and exploration of the mechanics is still useful. Although little has been done with SDS‐AuNPs, Alamadar et al. observed the interaction of SDS‐coated iron NPs with bacterial membranes. While no direct uptake was observed, visual observation via TEM imaging revealed interfacial surface activity.^[^ ^136^ ^]^ They additionally observed membrane destabilizing effects of the SDS‐coated NPs at higher concentrations, suggested to occur due to cell wall penetrating effects or oxidative stress.^[^ ^136^ ^]^ Similar interfacial activity and membrane destabilizing effects may be occurring in the current study. This aligns with Zhang et al. where the adsorption of silver NPs was shown to be either inhibited or promoted based on the localization of the NP organic matter coating.^[^ ^137^ ^]^ While coated NPs promoted adsorption, precoating the SLB with the organic matter inhibited adsorption.^[^ ^137^ ^]^ A similar process may be observed here, where coated SDS AuNPs promote adsorption, and unbound SDS interacting with the membrane may cause an inhibitory effect, allowing for alternative adsorption scenarios such as aggregation.

For TPA, the presence of the carboxylate groups could lead to distinct destabilizing effects during adsorption. Cao et al. have observed membrane disruption with ≈4 nm Au nanoclusters coated with thiol‐polyethylene glycol (PEG)‐carboxylic acid in bacterial cells, with the destabilization mechanism partially attributed to the protonation of carboxylate groups (leading to a change in membrane potential).^[^ ^138^ ^]^ This may explain the destabilization effects observed with TPA despite primarily interfacial bilayer activity.

In the CTAB system, electrostatics and AuNP‐ligand and ligand‐headgroup interactions may play a larger role in membrane destabilization and interfacial localization. Koch et al. report differences in adsorption between CIT‐AuNPs and CTAB‐AuNPs to polymersomes via DLS, suggesting the uptake of CTAB‐AuNPs is inhibited due to electrostatic repulsion.^[^ ^139^ ^]^ Peetla et al. had also observed the lack of adsorption of 130 nm CTAB‐coated polystyrene‐NPs on phosphocholine SLBs via AFM, and highlighted the primarily interfacial behavior.^[^ ^140^ ^]^ They observed similar behavior in other model phospholipid monolayers.^[^ ^141^ ^]^ While repulsive electrostatics may lead to greater adsorption inhibition rates, Shimokawa et al.^[^ ^142^ ^]^ observed both cationic and anionic NPs undergoing adsorption to bilayers if nonelectrostatic interactions such as vdW overcome electrostatics, and further note membrane deformation effects during adsorption due to electrostatic repulsion effects on charged lipids. A simultaneous adsorption and partial repulsion effect may be observed here that allows the incorporation of the CTAB AuNP partially within the membrane interface (potentially due to hydrophobic interactions), however, at the energetic cost of membrane distortion, leading to the lowered rupture force. The lack of adsorption may also be in part due to the inhibition of the dissociation of the ligand coating, which involves the balance between AuNP‐ligand surface attraction, as well as ligand dissociative capabilities of the SLB (through competitive surface interactions of the lipids on the AuNP surface). There is reportedly a strong association of the CTAB coating due to the self‐assembly of the energy minimum CTAB bilayer structure on the AuNP surface,^[^ ^143^ ^]^ so disassociation of the cap may require substantial work for there to be sufficient interaction between the AuNP and the lipid bilayer for subsequent adsorption, hence the interaction being localized to the interface. Additionally, CTAB‐AuNPs have been shown to be interfacial bilayer “tags” and remain arrested at a biomimetic phosphocholine‐containing liposomal interface.^[^ ^144^ ^]^ The apparent lack of clustering of the AuNPs on the SLB may be attributed to electrostatic repulsion.

Overall, these results highlight that ligand composition heavily influences NP adsorption behavior and can present a variety of different adsorption modes and aggregation behavior based on ligand composition, leading to various membrane destabilization and reorganization effects.

Collectively, the AFM results show that smaller ligand coatings, such as CIT, facilitate higher adsorption depths into the SLB, whereas larger ligand coatings, such as TAN, SDS, and TPA, localize the AuNP interaction to the bilayer headgroup region, and CTAB significantly inhibits AuNP from undergoing adsorption. These observations highlight the crucial role of ligand composition in the overall AuNP‐SLB adsorption process. In addition, through comparison of height versus rupture force profiles, the adaptation of AFM to measure differences in the adsorption mechanisms between different capped AuNPs is illustrated. While AFM is ideal for imaging changes to SLB integrity and structure, large‐scale aggregation behavior of AuNPs, and investigating the adsorption of single particles and the surrounding membrane area with nanoscale resolution, AFM cannot fully resolve dynamic atomistic detail of the NP‐SLB interaction. Therefore, MD simulations were employed as a complementary technique in order to understand these interactions in more detail.

### 2.2 MD Simulations

**Figure 3:**
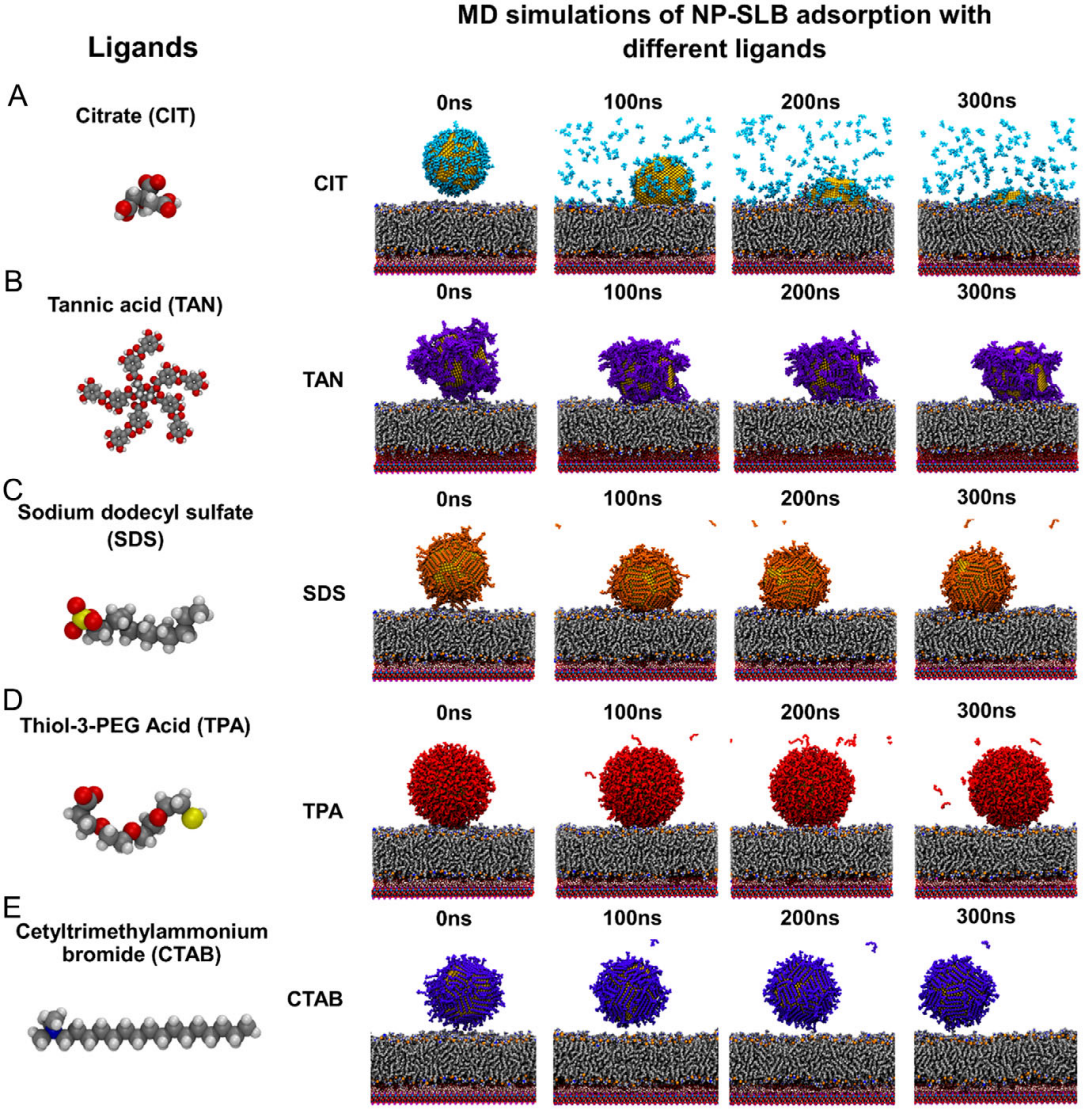
MD simulation of DOPC‐SLB systems interacting with NPs with various coatings of A) citrate (CIT), B) tannic acid (TAN), C) sodium dodecyl sulphate (SDS), D) thiol‐3‐PEG Acid (TPA), and E) cetyltrimethylammonium bromide (CTAB).

All‐atom MD simulations were employed to gain atomistic insight into adsorption/interaction mechanisms of the systems that were investigated experimentally (see **Figure** **3**). Components were chosen to represent the in situ AuNP‐SLB environment. The simulated systems include a single basal plane 15 nm × 15 nm of mica, a DOPC lipid bilayer, ≈1 nm water solvation layer in between the lipid bilayer and the mica, as well as the solvated AuNP‐ligand capped systems, with the capped systems being either CIT, TAN, SDS, TPA, or CTAB, denoting their AuNP capping agent. Additional baseline, non‐mica‐supported (floating) membranes can also be seen in Figure S8, Supporting Information. Based on previously benchmarked work,^[^ ^107^ ^]^ the capping agents were equilibrated in order to reach surface coverage densities between 90% and 100% to allow complete single monolayers of the ligands. **Table** **2** shows the ligand surface coverage densities on the AuNP in the simulations compared to experimental data. Notably, the MD surface coverage is always lower than that determined by experiment. These differences are probably due to the fact that in the simulation only a single layer is adsorbed, whereas in experiments the ligand shells may consist of double or multiple layers, clusters, etc.), and the experimental determination of this thickness is difficult in any case.

**Table 2:** Ligand and ligand‐capped AuNP characteristics and behavior.

Ligand	Charge	MD Surface Coverage	Experimental Surface Coverage	Behavior in AFM	Behavior in MD simulations
CIT	−1	157	218^[^ ^201^, ^202^ ^]^	Absorption	Absorption
TAN	0	39	242^[^ ^203^ ^]^	Interfacial adsorption	Interfacial adsorption
SDS	−1	119	348a) ^[^ ^204^ ^]^	Interfacial adsorption	Interfacial adsorption
TPA	−1	230	609^[^ ^205^ ^]^	Interfacial adsorption	Interfacial adsorption
CTAB	+1	125	158^[^ ^206^ ^]^	Occasional adsorption	No adsorption

a) Literature could not be found for specific SDS‐AuNP surface density, so the SDS value is based on a minimum hydrodynamic radius of a single SDS molecule vs AuNP surface area, considered to maximum surface density limit. (AuNP surface area/minimum SDS hydrodynamic radius = 121.7 nm^−2^/0.35 nm^−2^ = 347.71 SDS ligands per AuNP).

The systems can be divided into two primary components: the mica‐DOPC lipid membrane (SLB) system with the additional water solvation layer, and the solvated ligand‐capped AuNP systems. The pure DOPC‐SLB was equilibrated in isolation to form a baseline for analysis (see Figure S9, Supporting Information) and was commensurate with known fluid phase DOPC membrane models. The complete systems for analysis consisted of the equilibrated SLB membrane system and the equilibrated AuNP‐ligand systems. Adjustments to the pairwise nonbonded interactions between gold and the other atoms were made to reproduce the experimental coverage and exchange mechanisms (Table S1, Supporting Information).^[^ ^107^ ^]^ MD simulations of the ligand‐capped SLB systems were run for 300 ns with the ligand‐capped AuNP allowed to freely move within the system and the ligands not locked to the AuNP, allowing for their displacement as a function of interactions with the chemical environment over time (see Figure S10, Supporting Information for radial distribution functions (RDFs) of systems). An additional simulation was run as a bare nonligand capped AuNP for behavior comparison and can be found in Figure S11, Supporting Information.

**Figure 4:**
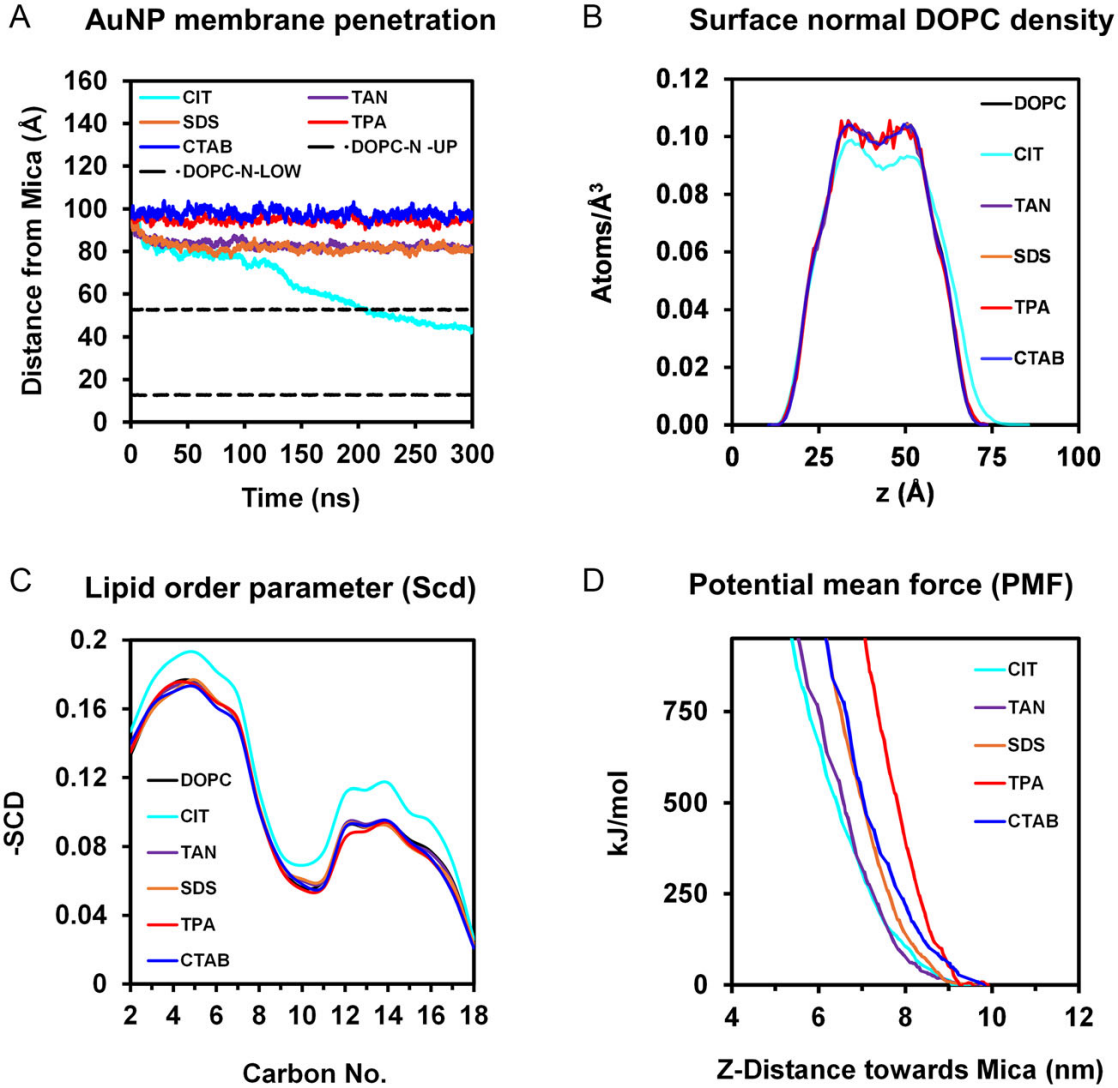
A) AuNP‐membrane *Z*‐axis penetration distance. Horizontal dashed black lines are representative of outer DOPC‐N headgroup positions. B) Surface normal DOPC density. C) lipid order parameter (*S*_cd_), and D) PMF for the ligands shown.

Figure 3 shows visual snapshots of the interactions for each of the ligands. The lower molecular weight (MW) CIT ligand facilitated sufficient hydrophobic contact between the AuNP surface and the lipids and enabled adsorption of the AuNP into the SLB. The larger MW ligands TAN, SDS, TPA, and CTAB enabled higher degrees of stable surface coverage, primarily shielding the interaction between the AuNP and the phospholipids, leading to incomplete adsorption. However, for TAN, SDS, and TPA, the ligand morphologies resulted in sufficient interfacial interaction with the headgroups, whereas with CTAB, the interaction was primarily inhibited due to electrostatic repulsion. CIT demonstrated the strongest bilayer interaction and underwent near complete adsorption into the membrane, showing progressive displacement of the CIT ligand coating as a function of lipid tail interactions and AuNP adsorption into the SLB. SDS and TAN, on the other hand, underwent interaction with the headgroups without ligand displacement or adsorption. Whereas TPA largely interacted with the outermost interface and CTAB was electrostatically inhibited from interacting with the SLB and sat close to the surface. These differences can be quantified via the penetration depth into the membrane. **Figure** **4**A shows the Z‐distance of the variously capped AuNPs’ center of mass relative to the mica surface oxygens over the 300 ns simulation. Only the CIT (cyan line) system shows adsorption past the nominal DOPC headgroups and reaches an equilibrium within the bilayer core at roughly ≈250 ns, while SDS and TAN are seen to reach an equilibrium distance from the headgroup region, TPA with the outermost headgroup region, and CTAB equilibrating slightly above the interface. As the CIT was able to undergo adsorption into the SLB, the CIT further showed significant changes to the membrane mechanics. Figure 4B shows the DOPC density for the final 10 ns of the simulation. The CIT‐SLB shows a distinct reduction in the central lipid density coupled to an increase in density at larger z‐value as the AuNP displaces the DOPC, whereas the SDS, TAN, TPA, and CTAB showed negligible changes as compared to baseline due to their lack of adsorption. Mechanical changes were further demonstrated by the lipid order parameter (*S*_cd_), which is a measure of the average lipid molecule orientation, with a value of ±0.5 corresponding to C—H bond alignment perpendicular to the bilayer surface and deviations from ±0.5 associated with increasing disorder. The *S*_cd_ in Figure 4C indicates a stiffening of the lipid chain during CIT adsorption, not observed for the SDS, TAN, TPA, and CTAB systems. This increased order exhibited by the lipid chains in the CIT system can be attributed to the compression caused by the reduction in available volume as the AuNP penetrates the membrane. Generalized disturbance to the membrane thickness and area per lipid (APL) because of this inclusion can also be observed (see Figure S12, Supporting Information). Additional properties such as leaflet interdigitation (see Figure S13, Supporting Information) show negligible changes, whilst the adsorption coincides with an increase in contact area (see Figure S14, Supporting Information).

Figure 4D shows umbrella sampling data of the AuNP‐capped systems (as shown in Figure 3A–E), as a function of the AuNP being pulled into the membrane via a biasing potential and reveals differences in the free energy profiles between the systems. Similar to the findings obtained from the unbiased MD simulation, the CIT‐AuNP underwent adsorption more easily compared to the other capped systems, with lower free energies indicative of easier adsorption within the membrane. In contrast, ligands that showed interfacial adsorption or no adsorption into the membrane (i.e., the TAN, SDS, TPA, and CTAB) had the highest potential of mean force (PMF) to incorporate the AuNP complex into the bilayer. It should be noted that the capping agents remained on the AuNP surface during the steered MD trajectory, except in the case of citrate which showed slight cap displacement, albeit to a lesser degree compared to the unbiased simulation. In the case of TPA (the system with the highest PMF gradient), the presence of the ether groups increases the chain hydrophilicity of the hydrocarbon chain, which increases the resistance of movement through the hydrophobic membrane core, resulting in a higher PMF gradient. The SDS‐capped and CTAB‐capped systems had the second highest PMF, as they are more easily incorporated into the bilayer than TPA due to their hydrophobic chains. However, there is still a minor energetic barrier due to their functional group‐headgroup repulsion interactions. The PMF of the citrate is reduced due to the transient nature of the cap and subsequent hydrophobic interaction between the AuNP and the lipid carbon tails. Van Lehn et al. observed increased adsorption of AuNPs at bilayer defects, specifically due to interactions between AuNPs and lipid tail protrusions.^[^ ^145^ ^]^ Interestingly, CIT and TAN have similar PMF profiles, despite their stark differences in ligand morphology/composition. In the case of CIT, the ligand‐facilitated interactions between the AuNP and lipid protrusions lowered the energetic cost for adsorption. Conversely, in TAN, the increased globular size of the ligand increased contacts with the lipid tail protrusions and similarly lowered the PMF profile. However, additional factors in the unbiased simulation, such as steric hindrance effects, still lead to inhibition of TAN adsorption due to the inability of cap dissociation. Additionally, similarities in the PMF of TAN and CIT may also further be attributed to TAN having both hydrophilic and hydrophobic components, as this may lower the energetic barrier into both the headgroups (hydrophilic) and bilayer core (hydrophobic). Su et al. had observed similar findings in relation to an optimum hydrophobic/hydrophilic compositional ratio of NPs that balances the attraction to the core and repulsion from headgroups to enable maximum translocation as it is not biased toward either region,^[^ ^146^ ^]^ potentially explaining the similar lowered PMF profile.

The observed differences in adsorption can be attributed to underlying differences in their mechanisms of action. The mechanism of adsorption of CIT‐AuNP with a DOPC‐SLB has been established in detail in previously published work.^[^ ^107^ ^]^ The adsorption process primarily involves the initial attraction, both vdW and electrostatic, between the negative citrate and the outer positive trimethylammonium lipid headgroups. This interaction enables proximal hydrophobic contact between the AuNP and the DOPC lipids. Subsequently, electrostatic repulsion of the citrate by the negatively charged phosphate groups of the DOPC lipid results in the dissociation of the ligand shell and adsorption of the AuNP.^[^ ^107^ ^]^

In contrast to the CIT, the TAN showed equilibrium within the headgroup interfacial region of the SLB and showed no deep adsorption into the membrane. It should be noted that the TAN changes the morphology of the AuNP complex more substantially than the other ligands, where the other ligands tend to conform to the shape of the AuNP surface. TAN forms unique protruding structures at the AuNP surface vertices due to the large size of the molecule, and as such, structural effects such as steric hindrance may be more apparent within this system. The TAN also shows negligible ligand dissociation, which can be explained by the strong hydrophobic association of the TAN ligand aromatic rings with the AuNP surface, inhibiting its dissociation and shielding the AuNP from interactions with the bilayer The interfacial adsorption is then explained by initial interaction between the trimethylamine methyl groups and the aromatic benzene rings, as well as the steric hindrance imparted by the large globular morphology of the tannic acid complex on the AuNP surface increasing the energetic barrier for complete adsorption.

Similar to the TAN system, the SDS also underwent interfacial interactions primarily within the headgroup region. The interfacial adsorption can be partially explained by the electrostatic interaction between the negatively charged sulfate functional groups and the positively charged trimethylamine DOPC headgroups. While specific examination of SDS‐coated AuNPs is lacking in the literature, approximation of the behavior of the NP may also be partially inferred from SDS's native properties. The strong hydrophobic interaction between the SDS carbon chain and the surface of the AuNP inhibits dissociation and allows it to remain strongly attached to the surface during membrane interfacial interaction, causing inhibition of adsorption of the AuNP into the SLB. Essentially, the presence of the cap shields the interactions between the AuNP and the SLB. Furthermore, while there is an attraction between the sulfate groups and the outer positive trimethylamine groups, the AuNP cannot absorb fully as desorption of SDS from the AuNP is energetically unfavorable, as well as the electrostatic repulsive barrier imposed on the negative sulfate groups of SDS molecules by the phosphate lipid headgroups.

Unlike the SDS and TAN systems, the TPA can be seen to localize at the top of the interface, as opposed to being embedded slightly within the headgroup region. The lack of further adsorption past the outermost headgroup can be partially attributed to the strongly adsorbed nature of the TPA ligands on the AuNP surface, inhibiting their ability to dynamically fluctuate.^[^ ^147^, ^148^, ^149^, ^150^, ^151^, ^152^ ^]^ The negative charge of the carboxylate group only permits interaction with the positive outer trimethylamine functional groups and is repelled by the negatively charged phosphate groups of the DOPC lipid, furthermore the hydrophilic nature of the TPA carbon chain (due to ether groups) may increase the solvent interactions and may increase energetic cost for adsorption.

In contrast to the other systems, CTAB showed inhibition of adsorption. This can partially be explained by the strong hydrophobic interactions of the CTAB carbon chains with the AuNP surface, limiting their dissociation from the surface.^[^ ^153^ ^]^ Additionally, both CTAB and DOPC have a positively charged trimethylamine functional group, resulting in electrostatic repulsion and the CTAB equilibrating slightly above the interface. While not in constant direct physical contact, the particle remained at very close proximity to the SLB (≈1 nm).

## 3 Discussion

The AFM and the MD showed comparable results for the CIT system, where AuNP adsorption and subsequent membrane destabilization were observed. MD results support the AFM observations of the interfacial adsorption of the TAN‐AuNP SLB with the lack of z‐penetration distance, however, they differ in their overall measured effect on the bilayer integrity. In the case of TAN, larger NPs typically have higher energetic costs of adsorption/permeation due to their size.^[^ ^154^ ^]^ Tannic acid hydroxyl and carbonyl groups are hydrophilic and may promote interactions with the headgroups, but the benzene rings are primarily hydrophobic and inhibit the desorption of the tannic acid from the AuNP surface, as well as introducing an energetic barrier for adsorption past the headgroups, localizing the AuNP to the interface. Tannic acid also has potential reactive oxygen species (ROS)‐inducing mechanisms^[^ ^133^ ^]^ that may disrupt the bilayer, which could explain the measured bilayer disturbance in AFM, despite the lack of complete adsorption. While specific chemical changes are not modeled in a classical MD simulation, the significance of measuring the localization of the AuNP to the interface aids in explaining the observed AFM results. Similarly, processes such as lipid oxidation resulting in membrane disruption can be measured indirectly with AFM. Notably, Orlowski et al. report uptake in their JAWS II dendritic cell line for their 5, 24, and 58 nm tannic acid‐coated AuNPs; however, when specific uptake proteins were inactivated, the AuNPs showed increased interfacial with the cell membrane and inhibition of internalization occured, highlighting the favorable interfacial interaction with the lipid bilayer.^[^ ^155^ ^]^

Discrepancies between the AFM and MD for SDS may be explained by differences in the order of timescale or localized detergent effects imposed by the SDS molecules on the SLB observed within AFM and not MD. The detergent effects of SDS on phospholipid membranes have primarily been found to be due to SDS molecules causing lipid “flip‐flop” events within the membrane, however, this process can take roughly in the order of minutes to hours for complete micellization.^[^ ^156^, ^157^ ^]^ As the time for post‐AuNP equilibration was roughly ≈30 min, this allows for these events to be observed in AFM, timescales which are not accessible in MD.

MD also shows localization of TPA‐AuNPs above the interface, supporting the AFM observations. Like the other larger size ligands investigated here, the localization of the TPA‐AuNP to the interface may be in part explained by steric hindrance induced from the presence of the TPA monolayer and electrostatic repulsion between the carboxylate groups on the TPA and the negatively charged DOPC phosphate group. The lack of adsorption observed here for a TPA coating is commensurate with Wang et al. who observed a significant decrease in bilayer adsorption for their TPA (chemisorbed) coatings over their CIT (physiosorbed) coatings due to their lowered interfacial interaction in their coarse‐grained model.^[^ ^106^ ^]^

While the MD showed an inhibition of interaction between the CTAB and DOPC, the AFM revealed primarily surface interaction with occasional adsorption and subsequent membrane destabilization. The discrepancies between the AFM and MD results may be rationalized as a factor of time; the AFM results could show higher adsorption rates due to NP settling processes at timescales which are unachievable via MD simulations. CTAB‐coated NPs have been observed to undergo primarily interfacial adsorption to a bilayer,^[^ ^140^, ^141^ ^]^ suggested in part due to electric repulsion mechanisms inhibiting complete uptake. While the MD and the AFM potentially differ in their localization of the NP, the results together show a reduced tendency of the CTAB‐AuNP to incorporate into the bilayer as opposed to other systems, such as CIT. Additionally, the electrostatic repulsion mechanism observed in the MD between the CTAB and the DOPC lipids may explain the AFM results, which showed the surface devoid of any large clustering features, as the NPs may have a high or negligible settling rate due to electrostatics (see Figure 2). However, some AuNPs can still occasionally attach to the membrane due to either thermal motion, hydrophobic contact, or AuNP‐ligand surface defects (due to improper coating), lowering the overall adsorption energy between the AuNP and the SLB.

Combining both AFM and MD results shows how the adsorption of a 5 nm AuNP with a DOPC‐SLB can be modulated via alteration of the capping agent. The MD and AFM reveal similar overall trends of NP adsorption between various ligand systems, primarily the increased adsorption of the CIT‐capped AuNP into the bilayer, with subsequent membrane disruption, and the interfacial or inhibited interaction of the TAN, SDS, TPA, and CTAB systems. AFM and MD results align in the localization of the bilayer; however, the AFM shows increased bilayer disturbance effects even for interfacially adsorbed AuNPs as opposed to MD. This may be explained via additional chemical effects, aggregation behavior, or timescales that are not accessible via MD. AFM and MD provide complimentary information. AFM can elucidate the large‐scale physical membrane changes and AuNP behavior in a dynamic SLB environment, while MD can be used to explore the detailed molecular behavior of the lipids, AuNPs and ligands. The results gathered here provide a method of establishing the absorbance tendencies of various capped NPs to SLBs, which can be adapted to other NP‐ligand‐SLB systems.

**Figure 5:**
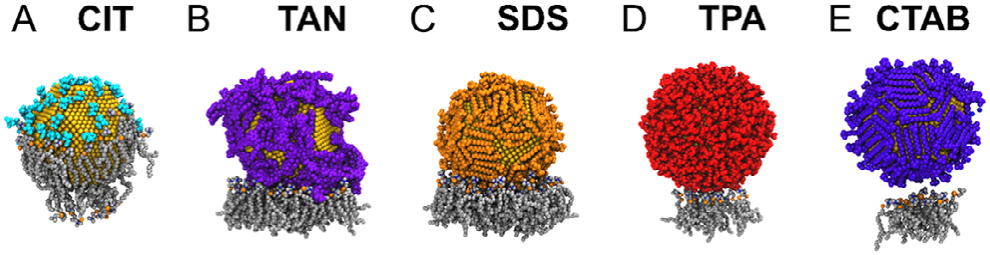
Close‐up isolated interfacial interaction snapshots during the adsorption/inhibition process, highlighting key interfacial interactions. A) CIT, bilayer adsorption, B) TAN, interfacial interaction, C) SDS, interfacial interaction, D) TPA, interfacial interaction, and E) CTAB, inhibition of adsorption.

Notably, the mechanistic behavior of the AuNP‐ligand complex observed within MD seems to be influenced by multiple factors, and it is difficult to isolate a single determinant for adsorption. Rather, it is the interplay of several ligand properties as well as their interactions with the AuNP and the membrane that dictate the specific adsorption behavior. While the adsorption of the AuNP was significantly influenced by ligand size, smaller‐sized ligands such as the CIT facilitated adsorption, and intermediate‐to‐large‐MW ligands such as the TAN, TPA, SDS, and CTAB showed incomplete adsorption, but size alone cannot explain the adsorption difference. Mid‐larger sized ligands of similar size, such as the TPA, SDS, and CTAB and TAN, showed differences in interfacial behavior. Electrostatic effects play a key role, such as in the case of SDS (negative) and CTAB (positive), where differences in functional groups lead to a difference in adsorption behavior. However, molecules with similar charge, such as the CIT (negative) and TPA (negative), showed differing adsorption behavior. Also, TAN (neutral) still showed interfacial adsorption, which highlights how additional ligand properties, such as morphology and hydrophobicity, must also play a role. Likewise, ligand chain hydrophobicity cannot fully explain the adsorption differences, as both CTAB and SDS chains are hydrophobic but display different adsorption behavior. **Figure** **5** presents a visual comparison of key headgroup interactions, indicating a multivariant mode of interaction determined by factors such as AuNP surface interactions, ligand hydrophobicity, solvent interactions, and geometric constraints. The ability to tailor different ligand behavior based on the coating composition remains apparent.

The observed ligand surface orientation and behavior are in line with the literature (see Table S2, Supporting Information, for a summary of AuNP‐ligand surface behavior). Citrate (CIT) ligands have been shown to form loosely bound transient monolayers on AuNP surfaces and be exchangeable with their solvent environment^[^ ^158^, ^159^, ^160^ ^]^ as well as undergo lipid–ligand exchange mechanisms in DOPC bilayers.^[^ ^103^, ^105^, ^106^ ^]^ For TAN, the formation of a globular network of tannic acid stabilized via vdW interactions around the AuNP with small protruding features (due to the large molecule size) is consistent with the observations by Walsh et al. of Au and aromatic rings, stabilized via noncovalent interactions and π–π stacking.^[^ ^161^ ^]^ For SDS, the hydrophobic adsorption of the tails on the Au surface with the headgroups facing outward in solution (see Figure S15C for RDF Au‐SDS terminal C vs Au‐SDS S), as described by Soares et al. is the energetically favorable configuration for SDS on Au surfaces.^[^ ^162^ ^]^ Thiolate ligands typically form Au–S surface bonds, which orient the R/tail group away from the surface.^[^ ^147^ ^]^ For TPA, a thiolate ligand, the Au–S vdW parameter (i.e., nonbonded interaction) was modeled according to the original interface parameters^[^ ^149^ ^]^ to represent their strong attractive nature.^[^ ^150^, ^151^, ^152^ ^]^ We observe a surface adsorbed S‐atom with the carboxylate sticking in solution (see Figure S15D, Supporting Information, RDF for Au‐TPA S and Au‐TPA carboxylate O), consistent with previous CG simulations by Lin et al.^[^ ^148^ ^]^ For CTAB, the hydrophobic adsorption of the carbon chains and the outward‐facing trimethylamine groups that associate with Br^−^ ions in solution is consistent with reports by Kalipillai et al. (see Figure S15E, Supporting Information, Au‐CTAB N and Au‐CTAB terminal C) who showed similar energetically favorable Au‐CTAB orientations.^[^ ^153^ ^]^

With respect to the presence of free ligands within the solution, ligand‐capped NPs, such as those used here, typically go through various purification and washing steps to remove excess ligands.^[^ ^163^ ^]^ Except for CIT, most of these ligands show minimal dissociation and favor the AuNP‐associated state. The presence of significant quantities of free ligands would result in the formation of micelles/aggregates which would be observable using DLS and AFM and their effects on the SLB (such as lipid detergent, oxidative, or destabilizing effects) would be apparent.^[^ ^133^, ^164^, ^165^, ^166^, ^167^, ^168^, ^169^, ^170^ ^]^ Additionally, as free ligands (like other exogenous molecules within the body) are typically rapidly cleared by the immune system,^[^ ^171^ ^]^ and as such, would not be present under realistic biological scenarios, this lowers the necessity of free ligand inclusion in the simulations.

In comparison to our CIT‐AuNP‐SLB system, recent experimental work by Contini et al. investigated the interfacial bilayer behavior of various‐sized citrate‐capped AuNPs with model DOPC liposomes.^[^ ^97^ ^]^ Notably, they observe spontaneous adsorption of their 5 nm AuNPs and rearrangement of the lipids, a wrapping of the membrane around the NP, and embedment of the AuNP within the bilayer.^[^ ^97^ ^]^ Cardellini et al. has furthered molecular investigation into the adsorption mechanisms by developing a coarse‐grain molecular model of the adsorption process of various‐sized (8 nm & 14 nm) citrate‐coated AuNPs onto a free‐floating DOPC membrane, where they observe the spontaneous adsorption of the AuNP, subsequent release of citrate molecules, and lipid re‐arrangement effects.^[^ ^105^ ^]^ Similar mechanisms of citrate‐capped AuNP adsorption to PC bilayers have been described elsewhere throughout the literature and are in line with the results gathered here.^[^ ^103^, ^104^ ^]^ The work here differs due to the use of all‐atomistic models and the use of the mica supporting mechanisms, however, parallels may be drawn in the overall adsorption process.

Work by Villanueva et al. also had similarities, involving the AFM investigation of the overall adsorption behavior of 2–5 nm CdTe quantum dots onto a mixed DOPC:DOTAP bilayer.^[^ ^172^ ^]^ They measure a drop in rupture force during NP‐SLB interaction. Additionally, while the initial interaction was facilitated via electrostatics, the hydrophobic attraction between the NP and the SLB was the determining factor for bilayer core incorporation.^[^ ^172^ ^]^ In relation to our interfacial adsorbed systems (i.e., SDS, TPA, CTAB, and TAN), recent experimental work by Wang et al. investigated the adsorption of various ligand‐coated 13 nm AuNPs onto a DOPC mixed lipid bilayer. Similar to observations here, the results show weakly physiosorbed ligands, such as citrate, enabled easier adsorption into the bilayer. Additionally, larger chemisorbed ligands such as thiolated ligands either did not directly adsorb or only weakly interacted with the SLB. This is in part due to the reduced exchangeability of the physiosorbed ligands with the solvent environment, whereas chemisorbed ligands remain on the surface and shield the interaction with the bilayer.^[^ ^106^ ^]^ These results are in line with our weakly adsorbed versus strongly adsorbed AuNP ligand observations and highlight the importance of ligand affinity in the overall NP‐SLB adsorption process.

Wang et al. used both computational modeling methods and AFM to support their findings. Our model builds upon this by establishing the adsorption mechanisms in the context of atomistic modeling, different size regimes, and alternative capping agents. Similar work by Lehn et al. also used MD and AFM for the analysis of AuNP bilayer adsorption and highlighted the significant role of the lipid tail interaction for the internalization of AuNPs.^[^ ^145^ ^]^ It was observed that AuNPs occasionally adsorb to the bilayer interface; however, in the presence of bilayer defects (such as lipid tail protrusions), AuNP insertion was primarily observed.^[^ ^145^ ^]^ That work highlights the advantages of combining AFM and MD techniques for the analysis of adsorption mechanisms, however, it does not give a comparison of ligand systems.^[^ ^145^ ^]^ Inhibiting bilayer adsorption through the alteration of the capping agent properties has been observed in a coarse‐grained model. On a mixed DPPC/DOPC bilayer, three modes of adsorption can be observed for their anionic, cationic, or hydrophobic/neutral AuNPs capping agents, where the AuNP can be seen to either localize to the headgroups, undergo absorption, or be inhibited from adsorption. This further highlights the interplay between ligand functional group composition and subsequent adsorption behavior.^[^ ^173^ ^]^ Additional investigations of the free energy profiles of capped versus noncapped AuNPs showed that thiolated ligands significantly increase the energetic barrier for adsorption.^[^ ^174^ ^]^ We highlight here the importance of using both computational and experimental methods, which is somewhat lacking in the literature. For example, the MARTINI forcefield is popularly used for the adsorption of AuNPs,^[^ ^104^, ^105^, ^173^ ^]^ and studies using this forcefield have shown adsorption for a highly physiosorbed PEGylated AuNPs.^[^ ^175^ ^]^ However, this is contrary to experimental findings of the adsorption of AuNPs with strong physiosorbed coatings, which show interfacial interaction and lack of complete adsorption.^[^ ^106^ ^]^ Recent work in which computational and simulation methods were used to understand AuNP bilayer translation by Zolghadr et al. provides an explanation for this observation.^[^ ^176^ ^]^ Their investigations into AuNP adsorption to a DPPC/POPC bilayer highlighted the underestimation of the free energy barrier using the MARTINI model, which would account for the increased adsorption of specific NPs.^[^ ^176^ ^]^ These results highlight the need for more combined computational/experimental AuNP‐bilayer studies, which, while they do exist within the literature, remain in their minority.^[^ ^105^, ^176^ ^]^ Additionally, there exists an even smaller niche of combined experimental/computational methods specifically for SLBs.^[^ ^106^, ^110^ ^]^

## 4 Conclusion

In this work, we used a combination of AFM and MD to characterize the adsorption behavior of various ligand‐coated AuNPs, with differing structural properties, primarily morphology and functional groups, at a fluid‐phase DOPC SLB. We further developed a platform for investigating NP‐membrane interactions atomistically, by using techniques that operate on different spatiotemporal ranges. The large‐scale biophysical behavior observed in the AFM can be further rationalized by the molecular mechanics observed in the MD. The specific MD‐inspired creation of the in situ SLB AFM environment and the NP‐ligand complexes, as well as the comparison of the observations made between AFM‐MD can be utilized by other researchers for the analysis of a plethora of NP‐membrane related systems and behaviors; notably to a higher degree of accuracy as compared to unsupported MD bilayer systems or isolated AFM investigations (which is currently extremely typical). Using these methods, we show the potential strength of combined AFM‐MD experimental work for the holistic characterization of NP‐SLB behavior. We encourage others to adapt these methods for the development of their own systems, for their analyzes of specific biophysical NP‐SLB behavior, which will further aid in adding to the limited but increasing body of knowledge surrounding NP‐SLB interactions.

The AFM established a relationship between the observed particle heights and the resultant change to rupture force, with increases in the particle penetration depths linked to increases in bilayer fluidity. However, bilayer disruption mechanisms were also observed for interfacially interacting AuNPs, and MD was used to further elucidate different interactions based on ligand composition. The MD simulations were able to differentiate specific adsorption inhibition, adsorption, or absorption mechanisms. While initial interaction (i.e., adsorption) is primarily driven by vdW/electrostatic interactions, the complete absorption of the NP is further driven by the propensity of the ligand coating to dissociate from the AuNP surface, as well as the ability of the AuNP to make sufficient hydrophobic contacts with the bilayer. The combined AFM and MD results suggest that the adsorption of the AuNP is modulated by the properties of the capping agent and aid in the mechanistic understanding of NP‐SLB adsorption mechanisms, which remain a significant area of investigation due to their relation to natural cellular mechanics and implications within the biomedical field. These methods may in part be used as a screening tool for identifying specific ligand compositions that can promote/hinder NP‐SLB adsorption and allow for the more rational design of NP therapeutics. These methods further highlight the significance of combined experimental/computational methods as their various spatiotemporal scales can aid in understanding the NP‐SLB system at various levels.

## 5 Experimental Section

5.1

5.1.1

##### Lipid Preparation

Lipid solutions were prepared for AFM via the following protocol. DOPC lipids (Avanti Polar Lipids Inc., AL, USA) were rehydrated into ultrapure Milli‐Q water to a concentration of 1 mg mL^−1^. The solutions were then bath‐sonicated at 55 °C for 30 min and subjected to a freeze‐thaw cycle (chilled to −20 °C for 30 min and then bath‐sonicated again). At this stage, solutions appeared uniformly “milky”, which is known to indicate the formation of multilamellar vesicles.^[^ ^125^ ^]^ The solution was then extruded at least 21 (but always an odd number) times using a Mini‐Extruder kit (Avanti Polar Lipids Inc., AL, USA) with a 200 nm filter membrane (Avanti Polar Lipids Inc., AL, USA). This process forms small, unilamellar vesicles (SUVs). The solution was then diluted with 0.15 M NaCl to a concentration of 0.1 mg mL^−1^. All glassware and components were cleaned thoroughly by sonication with ultrapure water, then isopropyl alcohol, and water again for 10 min each prior to use.

##### AuNPs

All AuNPs were purchased from a commercial supplier, Nanopartz (https://www.nanopartz.com/). The particles were purified using differential centrifugation protocols as described by the manufacturer to ensure monodispersity, stability, and removal of residual reactants.

##### Atomic Force Microscopy

The SLB were formed for surface‐based investigations via the vesicle‐fusion at a freshly cleaved (refreshed) mica (muscovite) interface.^[^ ^36^, ^177^ ^]^ In brief, the respective solutions were pipetted onto a freshly cleaved mica substrate and left to equilibrate for 20 min prior to imaging. This facilitates the spontaneous fusion of SUVs to the mica substrate.^[^ ^36^, ^177^ ^]^ This process formed SLBs. Each system was studied using a Cipher ES atomic force microscope (Oxford Instrument, Asylum Research, Santa Barbara, CA, USA) at room temperature (25 °C) using AM‐AFM. All images and force data were obtained using BioLever Mini BL‐AC40TS cantilevers (Oxford Instrument, Asylum Research, Santa Barbara, CA, USA, nominal spring constant kc = 0.09 N m^−1)^. To minimize the imaging force, a set point ratio (imaging amplitude (A)/free amplitude (A0)) of >0.7–0.8 was maintained, which has been shown to minimize any tip–sample distortion and damage for a variety of materials.^[^ ^178^, ^179^, ^180^, ^181^, ^182^ ^]^ Each cantilever was calibrated prior to use via the thermal spectrum method. Liquid experiments were completed in a droplet of lipid solution. This droplet was exposed to the atmosphere within an acoustic isolation cabinet (a sealed enclosure).

##### Molecular Dynamics Simulations

The 15 × 15 nm mica structure was constructed using the nanomaterial modeler on CHARMM‐GUI.^[^ ^183^, ^184^, ^185^ ^]^ The 5 nm AuNP was constructed using the Wulff Construction function of CHARMM‐GUI Nanomaterial Modeler, with a 2.5 nm radius and Miller indices 100, 110, and 111.^[^ ^183^, ^184^, ^185^ ^]^ The 15 × 15 nm DOPC membrane was constructed using the Membrane Builder of CHARMM‐GUI.^[^ ^186^ ^]^

Ligands were equilibrated around the AuNP in a 10 × 10 nm box containing 1 AuNP. There were 250 ligands for tannic acid, 500 ligands for CIT, CTAB, and SDS, and 750 for TPA. Solvated in 150 mM NaCl for 100 ns, ligands found within 0.5 nm of the AuNP surface were retained. Each complete system consisted of a 1 nm thick (15 nm^2^ laterally) layer of mica, a solvation layer to reproduce appropriate in situ membrane dynamics, the AuNP consisting of 4753 atoms and a surrounding ligand shell, solvated in a CHARMM TIP3P water box with 0.15 M NaCl and appropriate counter ions. Simulations were run using the MD code GROMACS 2023.^[^ ^187^ ^]^ Adapted from previous work, certain adjustments were made to the baseline structure files and forcefield parameters to match known literature behavior, including the removal of the potassium position restraints on the mica, to allow for the dissociation of K+ ions.^[^ ^188^, ^189^ ^]^ As well as the modulation of Au‐epsilon parameter to 3.5 for all pairwise interactions (except for Au–Au and Au–S for TPA) to accurately represent literature ligand coating coverage values (additional information Table S1, Supporting Information). Ligand topology and forcefield parameters for CIT, TPA, and TAN were assigned using ParamChem 2.4.0 CGenFF 4.4,^[^ ^190^, ^191^, ^192^ ^]^ whereas CTAB and SDS were assigned according to the CHARMM36 FF. It should also be noted that, since bromine is not included in CHARMM36, the bromine sigma/epsilon parameters were sourced from the CHARMM general forcefield. Horinek et al. had investigated ion forcefields using these bromine parameters^[^ ^193^ ^]^ and have comparable results to experimental properties (such as solvation energies). Storm et al. had similarly used these parameters and have a good recreation of CTAB micelle behavior.^[^ ^194^ ^]^ The interface CHARMM forcefield was applied to all atoms.^[^ ^149^, ^195^, ^196^ ^]^ While more recent water models allowed for better reproduction of bulk water dynamics, the TIP3P water model was used due to the compatibility between the CHARMM membrane model and the INTERFACE‐CHARMM nanomaterials.

Short‐range nonbonded interactions were treated using the Verlet cutoff scheme at a distance of 12 Å and a switch distance of 10 Å. Long‐range Coulomb interactions were calculated via the particle mesh Ewald method at a distance beyond 12 Å. The reference temperature during equilibration was 303.15 K and was controlled via the Berendsen temperature coupling algorithm. The temperature during the production run was 303.15 K and was controlled via Nosé–Hoover weak coupling dynamics, allowing for an canonical (NVT) ensemble, where the number of particles (N), volume (V) and temperature (T) and kept constant. A wall was implemented in the z‐dimension to prevent periodic self‐interactions. A time step of 2 fs was used with atomic coordinates saved every 10 ps. The full production run settings can be found in Table S3, Supporting Information.

After the SLB systems (DOPC–mica) were run in isolation for 100 ns to reach thermal equilibrium, the 5 nm AuNP‐ligand complexes were then added and run in triplicate for an additional 300 ns. All other simulation settings not listed here were the default settings generated by CHARMM‐GUI. The primary analysis was achieved via the use of GROMACS gmx analysis tools,^[^ ^187^ ^]^ MEMBPLUGIN^[^ ^197^ ^]^ in visual molecular dynamics (VMD),^[^ ^198^ ^]^ and FATSLiM.^[^ ^199^ ^]^ The main characteristics analyzed were the AuNP z‐diffusion, lipid carbon chain order parameters (*S*_cd_), lipid density function, and potential of mean force. Additional characteristics in the supporting information include the APL, membrane thickness, lipid interdigitation, radial distribution, and AuNP‐DOPC contact area.

##### Steered MD Simulations and Umbrella Sampling

Pulling simulations were performed for 1000ps, with a spring constant of 2000 KJ mol^−1^ and a pull rate of 0.01 nm ps^−1^ in GROMACS. The configurations from these simulations were subsequently used as input for umbrella sampling, weighted histogram analysis method was then applied to combine data from each sampling window to generate the PMF profiles.

##### DLS Characterization

DLS experiments were performed on an ALV‐5022 F light scattering spectrometer equipped with a laser wavelength of 633 nm. Measurements were taken in a cylindrical glass cuvette (inner diameter: 8 mm) (LSI Instruments, Fribourg) held in a scattering vat at room temperature.

##### TEM Characterization

TEM images were acquired using a JEOL1010 (JOEL, Musashino, Akishima, Tokyo, Japan) equipped with a JEOL 1010 TEM (2001) with a Gatan Orius SC600 CCD Camera (2014) and operated at an acceleration voltage of 200 keV. Images were processed and analyzed using Digital Micrograph 2.31.

##### Statistical Analysis

Atomic force microscopy imaging data is represented in raw form. Force spectroscopy and particle height were measured on *n* = 50 particles and presented as (mean ± SD). DLS size was reported as intensity‐weighted distributions (*n* = 3, repeats). TEM particle size distribution (*n* = 50) was presented as (mean ± SD), and cluster analysis was reported as distribution across population size (*n* = 50). MD simulations were run in triplicate (*n* = 3). MD simulations yielded Z‐penetration (raw), surface normal density (mean), lipid order parameter (*S*_cd_) (mean), potential mean force (raw), thickness (raw), APL (raw), RDF (raw), interdigitation (raw), Au‐contacts (raw), Au solvent accessible surface area (raw). No statistical testing was applied to AFM, DLS, TEM, or MD analysis. AFM data were processed via Gwyddion,^[^ ^200^ ^]^ Asylum research, and Igor pro as previously outlined.^[^ ^107^ ^]^ DLS was analyzed using the AVL correlator software, TEM was analyzed using Gatan and Gwyddion software. MD simulations were run using the MD code GROMACS 2023.^[^ ^187^ ^]^ MD analysis was undertaken using VMD,^[^ ^198^ ^]^ custom.tcl script for Z‐penetration. VMD native RDF or g(r) to calculate RDFs. MEMBPLUGIN^[^ ^197^ ^]^ was used to analyze the surface normal density, lipid order parameter, and interdigitation. GROMACS gmx analysis tools were used for analysis of PMF, Au‐contacts, and Au solvent accessible surface area.^[^ ^187^ ^]^ FATSLiM was used to analyze the APL and thickness measurements.^[^ ^199^ ^]^

## Conflict of Interest

The authors declare no conflict of interest.

## Author Contributions

**Rashad Kariuki**: conceptualization (lead); data curation (lead); formal analysis (lead); investigation (lead); methodology (lead); resources (lead); software (lead); validation (lead); visualization (lead); writing—original draft preparation (lead); writing—review and editing (lead). **Rowan Penman**: conceptualization (equal); data curation (equal); formal analysis (equal); investigation (equal); methodology (equal); resources (equal); software (equal); validation (equal); visualization (equal); writing—original draft preparation (lead); writing—review and editing (equal). **Alexander D. Newbold**: conceptualization (equal); data curation (equal); formal analysis (equal); investigation (equal); methodology (equal); resources (equal); software (equal); validation (equal); visualization (equal); writing—original draft preparation (lead); writing—review and editing (equal). **Kalpani A. Mirihana**: conceptualization (equal); data curation (equal); formal analysis (equal); investigation (equal); methodology (equal); resources (equal); software (equal); validation (equal); visualization (equal); writing—original draft preparation (lead); writing—review and editing (equal). **Pierre H. A. Vaillant**: conceptualization (supporting); data curation (supporting); formal analysis (supporting); investigation (supporting); methodology (supporting); resources (supporting); software (supporting); validation (supporting); visualization (supporting); writing—original draft preparation (supporting); writing—review and editing (supporting). **Tilly P. Shepherd**: conceptualization (supporting); data curation (supporting); formal analysis (supporting); investigation (supporting); methodology (supporting); resources (supporting); software (supporting); validation (supporting); visualization (supporting); writing—original draft preparation (supporting); writing—review and editing (supporting). **Nastaran Meftahi**: conceptualization (supporting); data curation (supporting); formal analysis (supporting); investigation (supporting); methodology (supporting); resources (supporting); software (supporting); validation (supporting); visualization (supporting); writing—original draft preparation (supporting); writing—review and editing (supporting). **Gary Bryant**: conceptualization (supporting); data curation (supporting); formal analysis (supporting); investigation (supporting); methodology (supporting); resources (supporting); software (supporting); validation (supporting); visualization (supporting); writing—original draft preparation (supporting); writing—review and editing (supporting). **Kislon Voïtchovsky**: conceptualization (supporting); data curation (supporting); formal analysis (supporting); investigation (supporting); methodology (supporting); resources (supporting); software (supporting); validation (supporting); visualization (supporting); writing—original draft preparation (supporting); writing—review and editing (supporting). **Claudia Contini**: conceptualization (supporting); data curation (supporting); formal analysis (supporting); funding acquisition (supporting); methodology (supporting); resources (supporting); software (supporting); validation (supporting); visualization (supporting); writing—original draft preparation (supporting); writing—review and editing (supporting). **Andrew Hung**: conceptualization (supporting); data curation (supporting); formal analysis (supporting); investigation (supporting); methodology (supporting); resources (supporting); software (supporting); validation (supporting); visualization (supporting); writing—original draft preparation (supporting); writing—review and editing (supporting). **Kevion K. Darmawan**: conceptualization (supporting); data curation (supporting); formal analysis (supporting); investigation (supporting); methodology (supporting); resources (supporting); software (supporting); validation (supporting); visualization (supporting); writing—original draft preparation (supporting); writing—review and editing (supporting). **Charlotte E. Conn**: conceptualization (supporting); data curation (supporting); formal analysis (supporting); funding acquisition (lead); methodology (supporting); project administration (lead); resources (lead); software (supporting); supervision (lead); validation (supporting); visualization (supporting); writing—original draft preparation (supporting); writing—review and editing (supporting). **Saffron J. Bryant**: conceptualization (lead); data curation (lead); formal analysis (lead); funding acquisition (lead); methodology (lead); project administration (lead); resources (lead); software (lead); supervision (lead); validation (lead); visualization (lead); writing—original draft preparation (lead); writing—review and editing (lead). **Andrew J. Christofferson**: conceptualization (lead); data curation (lead); formal analysis (lead); funding acquisition (lead); methodology (lead); project administration (lead); resources (lead); software (lead); supervision (lead); validation (lead); visualization (lead); writing—original draft preparation (lead); writing—review and editing (lead). **Aaron Elbourne**: conceptualization (lead); data curation (lead); formal analysis (lead); funding acquisition (lead); methodology (lead); project administration (lead); resources (lead); software (lead); supervision (lead); validation (lead); visualization (lead); writing—original draft preparation (lead); writing—review and editing (lead).

## Supporting information

Supplementary Material

## Data Availability

The data that support the findings of this study are available from the corresponding author upon reasonable request.

## References

[1] A. B. Asha , R. Narain , Polymer Science and Nanotechnology (Ed: R. Narain ), Amsterdam, Netherlands, Elsevier 2020, pp. 343–359.

[2] B. Mekuye , B. Abera , Nano Select 2023, 4, 486.

[3] Y.‐Y. Cai , Y. C. Choi , C. R. Kagan , Adv. Mater. 2023, 35, 2108104. 10.1002/adma.202108104 34897837

[4] L. Wang , M. Hasanzadeh Kafshgari , M. Meunier , Adv. Funct. Mater. 2020, 30, 2005400.

[5] B. Ni , G. Gonzalez‐Rubio , H. Cölfen , Acc. Chem. Res. 2022, 55, 1599. 35679581 10.1021/acs.accounts.2c00074

[6] U. Gupta , F. A. Escobedo , Langmuir 2022, 38, 1738. 35084868 10.1021/acs.langmuir.1c02804

[7] D. Lee , K. Huntoon , J. Lux , B. Y. S. Kim , W. Jiang , Nat. Rev. Bioeng. 2023, 1, 499.

[8] Z. Song , J. Fang , Z. Wang , R. Xiao , X. Guo , S. Zhou , Adv. Funct. Mater. 2023, 33, 2212326.

[9] X. Y. Wong , A. Sena‐Torralba , R. Álvarez‐Diduk , K. Muthoosamy , A. Merkoçi , ACS Nano 2020, 14, 2585. 32031781 10.1021/acsnano.9b08133

[10] X. Wu , H. Yang , W. Yang , X. Chen , J. Gao , X. Gong , H. Wang , Y. Duan , D. Wei , J. Chang , J. Mater. Chem. B 2019, 7, 4734. 31389961 10.1039/c9tb00860h

[11] S. Sarkar , B. Dyett , B. Lakic , A. S. Ball , L. Y. Yeo , J. F. White , S. Soni , C. J. Drummond , C. E. Conn , ACS Appl. Mater. Interfaces 2023, 15, 21819. 37018059 10.1021/acsami.3c00101

[12] J. Walther , D. Porenta , D. Wilbie , C. Seinen , N. Benne , Q. Yang , O. G. de Jong , Z. Lei , E. Mastrobattista , Eur. J. Pharm. Biopharm. 2024, 196, 114207. 38325664 10.1016/j.ejpb.2024.114207

[13] N. Dinauer , S. Balthasar , C. Weber , J. Kreuter , K. Langer , H. von Briesen , Biomaterials 2005, 26, 5898. 15949555 10.1016/j.biomaterials.2005.02.038

[14] L. G. Miranda Calderon , T. Alejo , S. Santos , G. Mendoza , S. Irusta , M. Arruebo , ACS Appl. Mater. Interfaces 2023, 15, 40213. 37596966 10.1021/acsami.3c07367 PMC10877563

[15] T. Wang , J. Bai , X. Jiang , G. U. Nienhaus , ACS Nano 2012, 6, 1251. 22250809 10.1021/nn203892h

[16] J. Sun , L. Zhang , J. Wang , Q. Feng , D. Liu , Q. Yin , D. Xu , Y. Wei , B. Ding , X. Shi , X. Jiang , Adv. Mater. 2015, 27, 1402. 25529120 10.1002/adma.201404788

[17] G. Caracciolo , S. Palchetti , V. Colapicchioni , L. Digiacomo , D. Pozzi , A. L. Capriotti , G. La Barbera , A. Laganà , Langmuir 2015, 31, 10764. 26378619 10.1021/acs.langmuir.5b02158

[18] K. Son , M. Ueda , K. Taguchi , T. Maruyama , S. Takeoka , Y. Ito , J. Contr. Release 2020, 322, 209. 10.1016/j.jconrel.2020.03.022 32194174

[19] H. Watson , Essays Biochem. 2015, 59, 43. 26504250 10.1042/bse0590043 PMC4626904

[20] H. Tao , T. Wu , M. Aldeghi , T. C. Wu , A. Aspuru‐Guzik , E. Kumacheva , Nat. Rev. Mater. 2021, 6, 701.

[21] N. Abid , A. M. Khan , S. Shujait , K. Chaudhary , M. Ikram , M. Imran , J. Haider , M. Khan , Q. Khan , M. Maqbool , Adv. Colloid Interface Sci. 2022, 300, 102597. 34979471 10.1016/j.cis.2021.102597

[22] R. Frost , S. Svedhem , C. Langhammer , B. Kasemo , Langmuir 2016, 32, 2708. 26907859 10.1021/acs.langmuir.5b03239

[23] N. Hoshyar , S. Gray , H. Han , G. Bao , Nanomedicine 2016, 11, 673. 27003448 10.2217/nnm.16.5 PMC5561790

[24] H. Abumanhal‐Masarweh , D. da Silva , M. Poley , A. Zinger , E. Goldman , N. Krinsky , R. Kleiner , G. Shenbach , J. E. Schroeder , J. Shklover , J. Shainsky‐Roitman , A. Schroeder , J. Contr. Release 2019, 307, 33. 10.1016/j.jconrel.2019.06.025 PMC6660975 31238049

[25] T. D. Brown , N. Habibi , D. Wu , J. Lahann , S. Mitragotri , ACS Biomater. Sci. Eng. 2020, 6, 4916. 33455287 10.1021/acsbiomaterials.0c00743

[26] M. M. Arnida , A. Ray , C. M. Peterson , H. Ghandehari , Eur. J. Pharm. Biopharm. 2011, 77, 417. 21093587 10.1016/j.ejpb.2010.11.010 PMC3379889

[27] T. Lunnoo , J. Assawakhajornsak , T. Puangmali , J. Phys. Chem. C 2019, 123, 3801. 10.1021/acs.jpcb.9b11600 32040917

[28] R. S. Hegde , R. J. Keenan , Nat. Rev. Mol. Cell Biol. 2022, 23, 107. 34556847 10.1038/s41580-021-00413-2 PMC12108547

[29] M. Moncan , K. Mnich , A. Blomme , A. Almanza , A. Samali , A. M. Gorman , J. Cell. Mol. Med. 2021, 25, 1359. 33398919 10.1111/jcmm.16255 PMC7875919

[30] C.‐P. Chng , K. J. Hsia , C. Huang , Soft Matter 2022, 18, 7752. 36093613 10.1039/d2sm00693f

[31] O. Zimmer , A. Goepferich , Nanoscale Horiz. 2023, 8, 256. 36594629 10.1039/d2nh00543c

[32] V. E. Debets , L. M. C. Janssen , A. Šarić , Soft Matter 2020, 16, 10628. 33084724 10.1039/d0sm00712a

[33] Y. Feng , Z. Kochovski , C. Arenz , Y. Lu , J. Kneipp , J. Phys. Chem. C 2022, 126, 13237. 10.1021/acs.jpcc.2c01930 PMC9377338 35983312

[34] S. J. Marrink , V. Corradi , P. C. T. Souza , H. I. Ingólfsson , D. P. Tieleman , M. S. P. Sansom , Chem Rev 2019, 119, 6184. 30623647 10.1021/acs.chemrev.8b00460 PMC6509646

[35] Y.‐H. M. Chan , S. G. Boxer , Curr. Opin. Chem. Biol. 2007, 11, 581. 17976391 10.1016/j.cbpa.2007.09.020 PMC2196400

[36] J. van Weerd , M. Karperien , P. Jonkheijm , Adv. Healthc. Mater. 2015, 4, 2743. 26573989 10.1002/adhm.201500398

[37] A. K. Rengan , M. Jagtap , A. De , R. Banerjee , R. Srivastava , Nanoscale 2014, 6, 916. 24281647 10.1039/c3nr04448c

[38] X. Ding , C. Yin , W. Zhang , Y. Sun , Z. Zhang , E. Yang , D. Sun , W. Wang , Nanoscale Res. Lett. 2020, 15, 68. 32232589 10.1186/s11671-020-03297-x PMC7105578

[39] F. Li , Q. Huang , Z. Zhou , Q. Guan , F. Ye , B. Huang , W. Guo , X.‐J. Liang , Signal Transduction Targeted Ther. 2023, 8, 285. 10.1038/s41392-023-01562-w PMC10393956 37528082

[40] A. Uzel , L. Agiotis , A. Baron , I. V. Zhigaltsev , P. R. Cullis , M. Hasanzadeh Kafshgari , M. Meunier , Small 2023, 19, 2305591. 10.1002/smll.202305591 37936336

[41] H. S. Kim , D. Y. Lee , Polymers 2018, 10, 961.30960886

[42] A. Jakhmola , T. K. Hornsby , F. M. Kashkooli , M. C. Kolios , K. Rod , J. Tavakkoli , Drug Delivery Trans. Res. 2024, 14, 2417. 10.1007/s13346-024-01516-x 38240946

[43] V. Thambi , A. Kar , P. Ghosh , D. Paital , A. R. S. Gautam , S. Khatua , ACS Omega 2019, 4, 13733. 31497690 10.1021/acsomega.9b01119 PMC6714510

[44] L. Panariello , S. Damilos , H. du Toit , G. Wu , A. N. P. Radhakrishnan , I. P. Parkin , A. Gavriilidis , React. Chem. Eng. 2020, 5, 663.

[45] G. Habibullah , J. Viktorova , T. Ruml , Nanoscale Res. Lett. 2021, 16, 47. 33721118 10.1186/s11671-021-03480-8 PMC7960878

[46] F. Gambinossi , S. E. Mylon , J. K. Ferri , Adv. Colloid Interface Sci. 2015, 222, 332. 25150615 10.1016/j.cis.2014.07.015

[47] N. William , F. Bamidoro , P. A. Beales , R. Drummond‐Brydson , N. Hondow , S. Key , A. Kulak , A. C. Walsh , S. Winter , L. A. Nelson , J. Colloid Interface Sci. 2021, 594, 101. 33756358 10.1016/j.jcis.2021.03.009

[48] J. R. Nicol , D. Dixon , J. A. Coulter , Nanomedicine 2015, 10, 1315. 25955125 10.2217/nnm.14.219

[49] K. Mahato , S. Nagpal , M. A. Shah , A. Srivastava , P. K. Maurya , S. Roy , A. Jaiswal , R. Singh , P. Chandra , 3 Biotech 2019, 9, 57. 10.1007/s13205-019-1577-z PMC6352626 30729081

[50] S. Chatterjee , X.‐Y. Lou , F. Liang , Y.‐W. Yang , Coord. Chem. Rev. 2022, 459, 214461.

[51] K. Shakerimanesh , F. Bayat , A. Shahrokhi , A. Baradaran , E. Yousefi , M. Mashreghi , A. Es‐Haghi , M. E. T. Yazdi , Mater. Technol. 2022, 37, 2853.

[52] V. S. Guido , P. H. M. L. OlivieriBrito Jr , M. L. Brito , B. C. Prezoto , D. S. T. Martinez , M. L. V. Oliva , A. A. Sousa , Langmuir 2024, 40, 12167. 38808371 10.1021/acs.langmuir.4c01123 PMC11171461

[53] B. B. Karakoçak , R. Raliya , J. T. Davis , S. Chavalmane , W.‐N. Wang , N. Ravi , P. Biswas , Toxicol. in Vitro 2016, 37, 61. 27599945 10.1016/j.tiv.2016.08.013

[54] Y. Wang , X. Li , Y. Wang , H. Chen , Y. Gao , Y. Lin , Mater. Des. 2024, 241, 112895.

[55] N. A. Alden , T. J. Yeingst , H. M. Pfeiffer , N. Celik , J. H. Arrizabalaga , A. M. Helton , Y. Liu , D. B. Stairs , A. B. Glick , N. Goyal , D. J. Hayes , Adv. Healthc. Mater. 2024, 13, 2303593. 38215360 10.1002/adhm.202303593 PMC11032112

[56] J.‐H. Yeom , E. Shin , H. Jin , H. Liu , Y. Luo , Y. Nam , M. Ryu , W. Song , H. Chi , J. Kim , K. Lee , J. Bae , J. Ind. Eng. Chem. 2023, 126, 480.

[57] W. Mao , H. S. Kim , Y. J. Son , S. R. Kim , H. S. Yoo , J. Contr. Release 2018, 269, 52. 10.1016/j.jconrel.2017.11.003 29113793

[58] L. Yao , D. Bojic , M. Liu , J. Pharm. Anal. 2023, 13, 960. 37842655 10.1016/j.jpha.2023.06.001 PMC10568098

[59] R. Zhang , F. Kiessling , T. Lammers , R. M. Pallares , Drug Delivery Trans. Res. 2023, 13, 378. 10.1007/s13346-022-01232-4 PMC9432795 36045273

[60] Y. Chen , J. Yang , S. Fu , J. Wu , Int. J. Nanomed. 2020, 15, 9407. 10.2147/IJN.S272902 PMC7699443 33262595

[61] Y. Wei , H. Chen , Y.‐X. Li , K. He , K. Yang , H.‐B. Pang , ACS Nano 2022, 16, 5885. 35302738 10.1021/acsnano.1c11068

[62] C. L. Ting , Z.‐G. Wang , Soft Matter 2012, 8, 12066.

[63] B. Molleman , T. Hiemstra , Phys. Chem. Chem. Phys. 2018, 20, 20575. 30059091 10.1039/c8cp02346h

[64] D. A. Perini , E. Parra‐Ortiz , I. Varó , M. Queralt‐Martín , M. Malmsten , A. Alcaraz , Langmuir 2022, 38, 14837. 36417698 10.1021/acs.langmuir.2c02487 PMC9974068

[65] F. Wu , Y. Tian , X. Luan , X. Lv , F. Li , G. Xu , W. Niu , Nano Lett. 2022, 22, 2915. 35362992 10.1021/acs.nanolett.2c00094

[66] D. Ling , M. J. Hackett , T. Hyeon , Nano Today 2014, 9, 457.

[67] K. Bhattacharjee , B. L. V. Prasad , Chem. Soc. Rev. 2023, 52, 2573. 36970981 10.1039/d1cs00876e

[68] Z. Zhang , W. Ma , K. He , B. Yuan , K. Yang , Phys. Chem. Chem. Phys. 2021, 23, 9158. 33885120 10.1039/d1cp00643f

[69] L. Sun , Y. Cao , X. Chen , Q. Liang , Commun. Theor. Phys. 2023, 75, 065601.

[70] C. A. Lochbaum , A. K. Chew , X. Zhang , V. Rotello , R. C. Van Lehn , J. A. Pedersen , ACS Nano 2021, 15, 6562. 33818061 10.1021/acsnano.0c09732 PMC9153949

[71] B. Stordy , Y. Zhang , Z. Sepahi , M. H. Khatami , P. M. Kim , W. C. W. Chan , Chem. Mater. 2022, 34, 6868.

[72] J. Broda , J. Setzler , A. Leifert , J. Steitz , R. Benz , U. Simon , W. Wenzel , Nanomed.: Nanotechnol., Biol. Med. 2016, 12, 1409. 10.1016/j.nano.2015.12.384 26773462

[73] L. Wang , P. Quan , S. H. Chen , W. Bu , Y.‐F. Li , X. Wu , J. Wu , L. Zhang , Y. Zhao , X. Jiang , B. Lin , R. Zhou , C. Chen , ACS Nano 2019, 13, 8680. 31329416 10.1021/acsnano.9b00114

[74] A. Kordzadeh , M. Zarif , S. Amjad‐Iranagh , Comp. Methods Prog. Biomed. 2023, 230, 107332. 10.1016/j.cmpb.2022.107332 36603233

[75] H. Y. Yang , Y. Li , D. S. Lee , Adv. NanoBiomed. Res. 2021, 1, 2000043.

[76] J.‐B. Fleury , V. A. Baulin , X. Le Guével , Nanoscale 2022, 14, 13178. 36043913 10.1039/d2nr01339h

[77] D. Gerstner , T. Kraus , Nanoscale 2018, 10, 8009. 29666855 10.1039/c8nr00597d

[78] B. M. Sperry , N. A. Kukhta , Y. Huang , C. K. Luscombe , Chem. Mater. 2023, 35, 570. 36711050 10.1021/acs.chemmater.2c03006 PMC9879203

[79] D. Pozzi , G. Caracciolo , L. Digiacomo , V. Colapicchioni , S. Palchetti , A. L. Capriotti , C. Cavaliere , R. Zenezini Chiozzi , A. Puglisi , A. Laganà , Nanoscale 2015, 7, 13958. 26222625 10.1039/c5nr03701h

[80] A. Perez‐Potti , H. Lopez , B. Pelaz , A. Abdelmonem , M. G. Soliman , I. Schoen , P. M. Kelly , K. A. Dawson , W. J. Parak , Z. Krpetic , M. P. Monopoli , Sci. Rep. 2021, 11, 6443. 33742032 10.1038/s41598-021-84029-8 PMC7979877

[81] T. Lima , K. Bernfur , M. Vilanova , T. Cedervall , Sci. Rep. 2020, 10, 1129. 31980686 10.1038/s41598-020-57943-6 PMC6981174

[82] L. L. Olenick , J. M. Troiano , A. Vartanian , E. S. Melby , A. C. Mensch , L. Zhang , J. Hong , O. Mesele , T. Qiu , J. Bozich , S. Lohse , X. Zhang , T. R. Kuech , A. Millevolte , I. Gunsolus , A. C. McGeachy , M. Doğangün , T. Li , D. Hu , S. R. Walter , A. Mohaimani , A. Schmoldt , M. D. Torelli , K. R. Hurley , J. Dalluge , G. Chong , Z. V. Feng , C. L. Haynes , R. J. Hamers , et al., Chem 2018, 4, 2709.

[83] A. J. Chetwynd , I. Lynch , Environ. Sci.: Nano 2020, 7, 1041.

[84] M. S. Jahan Sajib , P. Sarker , Y. Wei , X. Tao , T. Wei , Langmuir 2020, 36, 13356. 33124831 10.1021/acs.langmuir.0c02767

[85] C. Carnovale , G. Bryant , R. Shukla , V. Bansal , Prog. Mater. Sci. 2016, 83, 152.

[86] C. Carnovale , G. Bryant , R. Shukla , V. Bansal , ACS omega 2019, 4, 242.

[87] X. Lin , X. Lin , N. Gu , Nanoscale 2020, 12, 4101. 32022059 10.1039/c9nr09226a

[88] C.‐Y. Hsia , M. J. Richards , S. Daniel , Anal. Methods 2015, 7, 7076.

[89] M. M. Modena , B. Rühle , T. P. Burg , S. Wuttke , Adv. Mater. 2019, 31, 1901556. 10.1002/adma.201901556 31148285

[90] H. Gao , J. Mech. Phys. Solids 2014, 62, 312.

[91] T. Palacios‐Hernandez , D. M. Diaz‐Diestra , A. K. Nguyen , S. A. Skoog , B. Vijaya Chikkaveeraiah , X. Tang , Y. Wu , P. E. Petrochenko , E. M. Sussman , P. L. Goering , J. Appl. Toxicol. 2020, 40, 918. 32080871 10.1002/jat.3953

[92] X. Jia , S. Wang , L. Zhou , L. Sun , Nanoscale Res. Lett. 2017, 12, 478. 28774157 10.1186/s11671-017-2242-2 PMC5540742

[93] S. H. Tabari , Y. Jamali , R. Poursalehi , Procedia Mater. Sci. 2015, 11, 423.

[94] E. S. Melby , C. Allen , I. U. Foreman‐Ortiz , E. R. Caudill , T. R. Kuech , A. M. Vartanian , X. Zhang , C. J. Murphy , R. Hernandez , J. A. Pedersen , Langmuir 2018, 34, 10793. 30102857 10.1021/acs.langmuir.8b02060

[95] X. Xie , J. Liao , X. Shao , Q. Li , Y. Lin , Sci. Rep. 2017, 7, 3827. 28630477 10.1038/s41598-017-04229-z PMC5476625

[96] C. A. Huang‐Zhu , J. K. Sheavly , A. K. Chew , S. J. Patel , R. C. Van Lehn , ACS Nano 2024, 18, 6424. 38354368 10.1021/acsnano.3c11854 PMC11298871

[97] C. Contini , J. W. Hindley , T. J. Macdonald , J. D. Barritt , O. Ces , N. Quirke , Commun. Chem. 2020, 3, 130. 33829115 10.1038/s42004-020-00377-y PMC7610534

[98] C. Paba , V. Dorigo , B. Senigagliesi , N. Tormena , P. Parisse , K. Voitchovsky , L. Casalis , J. Colloid Interface Sci. 2023, 652, 1937. 37690301 10.1016/j.jcis.2023.08.117

[99] A. R. Mhashal , S. Roy , PLOS ONE 2014, 9, e114152. 25469786 10.1371/journal.pone.0114152 PMC4255040

[100] Q. Yu , S. Dasgupta , T. Auth , G. Gompper , Nano Lett. 2020, 20, 1662. 32046489 10.1021/acs.nanolett.9b04788

[101] J. Zhao , M. H. Stenzel , Polym. Chem. 2018, 9, 259.

[102] S. Zhang , H. Gao , G. Bao , ACS Nano 2015, 9, 8655. 26256227 10.1021/acsnano.5b03184 PMC5681865

[103] C. Montis , L. Caselli , F. Valle , A. Zendrini , F. Carlà , R. Schweins , M. Maccarini , P. Bergese , D. Berti , J. Colloid Interface Sci. 2020, 573, 204. 32278951 10.1016/j.jcis.2020.03.123

[104] S. Salassi , L. Caselli , J. Cardellini , E. Lavagna , C. Montis , D. Berti , G. Rossi , J. Chem. Theor. Comput. 2021, 17, 6597. 10.1021/acs.jctc.1c00627 PMC8515808 34491056

[105] J. Cardellini , L. Caselli , E. Lavagna , S. Salassi , H. Amenitsch , M. Calamai , C. Montis , G. Rossi , D. Berti , J. Phys. Chem. C 2022, 126, 4483. 10.1021/acs.jpcc.1c08914 PMC8919252 35299820

[106] X. Wang , X. Wang , X. Bai , L. Yan , T. Liu , M. Wang , Y. Song , G. Hu , Z. Gu , Q. Miao , C. Chen , Nano Lett. 2019, 19, 8. 30335394 10.1021/acs.nanolett.8b02638

[107] R. Kariuki , R. Penman , S. J. Bryant , R. Orrell‐Trigg , N. Meftahi , R. J. Crawford , C. F. McConville , G. Bryant , K. Voïtchovsky , C. E. Conn , A. J. Christofferson , A. Elbourne , ACS Nano 2022, 16, 17179. 36121776 10.1021/acsnano.2c07751

[108] M. Bekir , A. Hörmann , C. Brückner , I. Hoffmann , S. Prévost , M. Gradzielski , Langmuir 2021, 37, 2800. 33606547 10.1021/acs.langmuir.0c03553

[109] M. Zaborowska , A. Bartkowiak , E. Nazaruk , D. Matyszewska , R. Bilewicz , Langmuir 2023, 39, 7958. 37231652 10.1021/acs.langmuir.3c00810 PMC10249408

[110] T. Pfeiffer , A. De Nicola , C. Montis , F. Carlà , N. F. A. van der Vegt , D. Berti , G. Milano , J. Phys. Chem. Lett. 2019, 10, 129. 30563321 10.1021/acs.jpclett.8b03399

[111] L. Caselli , T. Traini , S. Micciulla , F. Sebastiani , S. Köhler , E. M. Nielsen , R. G. Diedrichsen , M. W. A. Skoda , M. Malmsten , Adv. Funct. Mater. 2024, 34, 2405047.

[112] F. Lolicato , L. Joly , H. Martinez‐Seara , G. Fragneto , E. Scoppola , F. Baldelli Bombelli , I. Vattulainen , J. Akola , M. Maccarini , Small 2019, 15, 1805046. 10.1002/smll.201805046 31012268

[113] S. Tatur , M. Maccarini , R. Barker , A. Nelson , G. Fragneto , Langmuir 2013, 29, 6606. 23638939 10.1021/la401074y

[114] N. Kanwa , A. Patnaik , S. K. De , M. Ahamed , A. Chakraborty , Langmuir 2019, 35, 1008. 30601000 10.1021/acs.langmuir.8b03673

[115] E. Blanco , H. Shen , M. Ferrari , Nat. Biotechnol. 2015, 33, 941. 26348965 10.1038/nbt.3330 PMC4978509

[116] A. Bhirde , J. Xie , M. Swierczewska , X. Chen , Nanoscale 2011, 3, 142. 20938522 10.1039/c0nr00493f PMC6454877

[117] L. Yuan , Q. Chen , J. E. Riviere , Z. Lin , J. Drug Delivery Sci. Technol. 2023, 83, 104404. 10.1016/j.jddst.2023.104404 PMC10686544 38037664

[118] F. Wang , J. Liu , Nanoscale 2015, 7, 15599. 26372064 10.1039/c5nr04805b

[119] Y. Jiang , S. Huo , T. Mizuhara , R. Das , Y.‐W. Lee , S. Hou , D. F. Moyano , B. Duncan , X.‐J. Liang , V. M. Rotello , ACS Nano 2015, 9, 9986. 26435075 10.1021/acsnano.5b03521 PMC5848075

[120] E. Canepa , S. Salassi , F. Simonelli , R. Ferrando , R. Rolandi , C. Lambruschini , F. Canepa , S. Dante , A. Relini , G. Rossi , Sci. Rep. 2021, 11, 1256. 33441958 10.1038/s41598-020-80953-3 PMC7807088

[121] M. V. Chiarpotti , G. S. Longo , M. G. Del Pópolo , J. Phys. Chem. B 2022, 126, 2230. 35293749 10.1021/acs.jpcb.1c10499

[122] A. Ridolfi , L. Caselli , C. Montis , G. Mangiapia , D. Berti , M. Brucale , F. Valle , J. Microsc. 2020, 280, 194. 32432336 10.1111/jmi.12910

[123] A. Alessandrini , P. Facci , Smart Membr. Sensors 2014, 185.

[124] A. R. Burns , D. J. Frankel , T. Buranda , Biophys. J. 2005, 89, 1081. 15879469 10.1529/biophysj.105.060327 PMC1366593

[125] W. Trewby , J. Faraudo , K. Voïtchovsky , Nanoscale 2019, 11, 4376. 30801089 10.1039/c8nr06339g

[126] C. A. Keller , K. Glasmästar , V. P. Zhdanov , B. Kasemo , Phys. Rev. Lett. 2000, 84, 5443. 10990964 10.1103/PhysRevLett.84.5443

[127] A. Åkesson , T. Lind , N. Ehrlich , D. Stamou , H. Wacklin , M. Cárdenas , Soft Matter 2012, 8, 5658.

[128] J. D. Unsay , K. Cosentino , A. J. García‐Sáez , J. Vis. Exp. 2015, e52867. 26273958 10.3791/52867 PMC4545161

[129] H. Tae , S. Park , S.‐O. Kim , S. Yorulmaz Avsar , N.‐J. Cho , J. Phys. Chem. B 2022, 126, 2345. 35316051 10.1021/acs.jpcb.1c10534

[130] S.‐J. Marrink , O. Berger , P. Tieleman , F. Jähnig , Biophys. J. 1998, 74, 931. 9533704 10.1016/S0006-3495(98)74016-0 PMC1302572

[131] E. J. Miller , K. Voïtchovsky , M. Staykova , Nanoscale 2018, 10, 16332. 30132496 10.1039/c8nr03399d

[132] X. Cui , S. Mao , M. Liu , H. Yuan , Y. Du , Langmuir 2008, 24, 10771. 18729337 10.1021/la801705y

[133] P. K. B. Nagesh , P. Chowdhury , E. Hatami , S. Jain , N. Dan , V. K. Kashyap , S. C. Chauhan , M. Jaggi , M. M. Yallapu , Sci. Rep. 2020, 10, 980. 31969643 10.1038/s41598-020-57932-9 PMC6976712

[134] B. K. Yoon , J. A. Jackman , M. C. Kim , N.‐J. Cho , Langmuir 2015, 31, 10223. 26325618 10.1021/acs.langmuir.5b02088

[135] P. Azcona , R. Zysler , V. Lassalle , Colloids Surf. A: Physicochem. Eng. Asp. 2016, 504, 320.

[136] N. Alamdar , B. Rasekh , F. Yazdian , IET Nanobiotechnol. 2018, 12, 520. 29768241 10.1049/iet-nbt.2016.0260 PMC8675947

[137] Z. Bo , S. Y. Avsar , M. K. Corliss , M. Chung , N.‐J. Cho , J. Hazard. Mater. 2017, 339, 264. 28654791 10.1016/j.jhazmat.2017.06.031

[138] Z. Cao , X. Chen , J. Chen , A. Xia , B. Bacacao , J. Tran , D. Sharma , L. A. Bekale , P. L. Santa Maria , Nanoscale 2022, 14, 10016. 35796201 10.1039/d2nr01003h PMC9578678

[139] A. H. R. Koch , S. Morsbach , T. Bereau , G. Lévêque , H.‐J. Butt , M. Deserno , K. Landfester , G. Fytas , J. Phys. Chem. B 2020, 124, 742. 31951417 10.1021/acs.jpcb.9b10469 PMC7008459

[140] C. Peetla , V. Labhasetwar , Langmuir 2009, 25, 2369. 19161268 10.1021/la803361y PMC2653596

[141] C. Peetla , S. Jin , J. Weimer , A. Elegbede , V. Labhasetwar , Langmuir 2014, 30, 7522. 24911361 10.1021/la5015219 PMC4079324

[142] N. Shimokawa , H. Ito , Y. Higuchi , Phys. Rev. E 2019, 100, 012407. 31499808 10.1103/PhysRevE.100.012407

[143] R. Li , Z. Wang , X. Gu , C. Chen , Y. Zhang , D. Hu , ACS Omega 2020, 5, 4943. 32201780 10.1021/acsomega.9b03823 PMC7081447

[144] H. Ba , J. Rodríguez‐Fernández , F. D. Stefani , J. Feldmann , Nano Lett. 2010, 10, 3006. 20614869 10.1021/nl101454a

[145] R. C. Van Lehn , M. Ricci , P. H. J. Silva , P. Andreozzi , J. Reguera , K. Voïtchovsky , F. Stellacci , A. Alexander‐Katz , Nat. Commun. 2014, 5, 4482. 25042518 10.1038/ncomms5482

[146] C.‐F. Su , H. Merlitz , H. Rabbel , J.‐U. Sommer , J. Phys. Chem. Lett. 2017, 8, 4069. 28797162 10.1021/acs.jpclett.7b01888

[147] M. K. Corbierre , R. B. Lennox , Chem. Mater. 2005, 17, 5691.

[148] J.‐Q. Lin , H.‐W. Zhang , Z. Chen , Y.‐G. Zheng , Z.‐Q. Zhang , H.‐F. Ye , J. Phys. Chem. C 2011, 115, 18991.

[149] H. Heinz , R. A. Vaia , B. L. Farmer , R. R. Naik , J. Phys. Chem. C 2008, 112, 17281.

[150] F. Tielens , E. Santos , J. Phys. Chem. C 2010, 114, 9444.

[151] M. S. Inkpen , Z. F. Liu , H. Li , L. M. Campos , J. B. Neaton , L. Venkataraman , Nat. Chem. 2019, 11, 351. 30833721 10.1038/s41557-019-0216-y

[152] L. B. Wright , P. M. Rodger , S. Corni , T. R. Walsh , J. Chem. Theor. Comput. 2013, 9, 1616. 10.1021/ct301018m 26587623

[153] P. Kalipillai , E. Raghuram , S. Bandyopadhyay , E. Mani , Phys. Chem. Chem. Phys. 2022, 24, 28353. 36385573 10.1039/d2cp02202h

[154] S. Erimban , S. Daschakraborty , J. Chem. Sci. 2021, 133, 105.

[155] P. Orlowski , E. Tomaszewska , K. Ranoszek‐Soliwoda , M. Gniadek , O. Labedz , T. Malewski , J. Nowakowska , G. Chodaczek , G. Celichowski , J. Grobelny , M. Krzyzowska , Front. Immunol. 2018, 9, 1115. 29872440 10.3389/fimmu.2018.01115 PMC5972285

[156] U. Kragh‐Hansen , M. le Maire , J. V. Møller , Biophys. J. 1998, 75, 2932. 9826614 10.1016/S0006-3495(98)77735-5 PMC1299965

[157] T. P. Sudbrack , N. L. Archilha , R. Itri , K. A. Riske , J. Phys. Chem. B 2011, 115, 269. 21171656 10.1021/jp108653e

[158] M. Rani , L. Moudgil , B. Singh , A. Kaushal , A. Mittal , G. S. S. Saini , S. K. Tripathi , G. Singh , A. Kaura , RSC Adv. 2016, 6, 17373.

[159] G. S. Perera , S. A. Athukorale , F. Perez , C. U. Pittman , D. Zhang , J. Colloid Interface Sci. 2018, 511, 335. 29031153 10.1016/j.jcis.2017.10.014

[160] H. Wei , W. Leng , J. Song , C. Liu , M. R. Willner , Q. Huang , W. Zhou , P. J. Vikesland , Environ. Sci. Technol. 2019, 53, 575. 30525495 10.1021/acs.est.8b03144

[161] J. P. Palafox‐Hernandez , C.‐K. Lim , Z. Tang , K. L. M. Drew , Z. E. Hughes , Y. Li , M. T. Swihart , P. N. Prasad , M. R. Knecht , T. R. Walsh , ACS Appl. Mater. Interfaces 2016, 8, 1050. 26684587 10.1021/acsami.5b11989

[162] D. M. Soares , W. E. Gomes , M. A. Tenan , Langmuir 2007, 23, 4383. 17348697 10.1021/la063508+

[163] A. M. El‐Khawaga , A. Zidan , A. I. A. A. El‐Mageed , J. Mol. Struct. 2023, 1281, 135148.

[164] M. Mura , B. Humphreys , J. Gilbert , A. Salis , T. Nylander , Colloids Surf. B: Biointerfaces 2023, 223, 113187. 36739672 10.1016/j.colsurfb.2023.113187

[165] V. A. Bjørnestad , R. Lund , Langmuir 2023, 39, 3914. 36893452 10.1021/acs.langmuir.2c03207 PMC10035035

[166] O. Garbuzenko , Y. Barenholz , A. Priev , Chem. Phys. Lipids 2005, 135, 117. 15921973 10.1016/j.chemphyslip.2005.02.003

[167] A. Mahendra , H. P. James , S. Jadhav , Chem. Phys. Lipids 2019, 218, 47. 30521788 10.1016/j.chemphyslip.2018.12.001

[168] J. Shen , Y. Wang , Q. Ping , Y. Xiao , X. Huang , J. Contr. Release 2009, 137, 217. 10.1016/j.jconrel.2009.04.021 19393270

[169] J. Wu , Y. Li , X. Chen , N. Li , W. He , Y. Feng , J. Liu , Sci. Total Environ. 2022, 822, 153443. 35092767 10.1016/j.scitotenv.2022.153443

[170] Y. S. Lai , Y. Zhou , E. Eustance , L. Straka , Z. Wang , B. E. Rittmann , Algal Res. 2018, 34, 250.

[171] B. G. Keselowsky , A. Acharya , J. S. Lewis , Biomaterials Science (Fourth Edition), (Eds: W. R. Wagner , S. E. Sakiyama‐Elbert , G. Zhang , M. J. Yaszemski ), London, UK, Academic Press 2020, pp. 747–775.

[172] M. E. Villanueva , L. Bar , L. Porcar , Y. Gerelli , P. Losada‐Pérez , J. Colloid Interface Sci. 2025, 677, 620. 39116560 10.1016/j.jcis.2024.07.220

[173] X. Quan , C. Peng , D. Zhao , L. Li , J. Fan , J. Zhou , Langmuir 2017, 33, 361. 27794619 10.1021/acs.langmuir.6b02937

[174] A. R. Mhashal , S. Roy , ChemPhysChem 2016, 17, 3504. 27595236 10.1002/cphc.201600690

[175] P. A. Oroskar , C. J. Jameson , S. Murad , Langmuir 2016, 32, 7541. 27399834 10.1021/acs.langmuir.6b01740

[176] A. R. Zolghadr , S. S. Moosavi , RSC Adv. 2019, 9, 5197. 35514645 10.1039/c8ra06777e PMC9060696

[177] Z. Lv , S. Banerjee , K. Zagorski , Y. L. Lyubchenko , Methods Mol. Biol. 2018, 1814, 129. 29956230 10.1007/978-1-4939-8591-3_8 PMC6045422

[178] P. D. R. García , Amplitude Modulation Atomic Force Microscopy 2010, pp. 77–90.

[179] K. Voïtchovsky , Phys. Rev. E 2013, 88, 022407. 10.1103/PhysRevE.88.022407 24032849

[180] A. J. Page , A. Elbourne , R. Stefanovic , M. A. Addicoat , G. G. Warr , K. Voïtchovsky , R. Atkin , Nanoscale 2014, 6, 8100. 24916188 10.1039/c4nr01219d

[181] A. Elbourne , S. McDonald , K. Voïchovsky , F. Endres , G. G. Warr , R. Atkin , ACS Nano 2015, 9, 7608. 26051040 10.1021/acsnano.5b02921

[182] A. Elbourne , B. McLean , K. Voïtchovsky , G. G. Warr , R. Atkin , The Journal of Physical Chemistry Letters 2016, 7, 3118. 27463824 10.1021/acs.jpclett.6b01323

[183] S. Jo , T. Kim , V. G. Iyer , W. Im , J. Comput. Chem. 2008, 29, 1859. 18351591 10.1002/jcc.20945

[184] Y. K. Choi , N. R. Kern , S. Kim , K. Kanhaiya , Y. Afshar , S. H. Jeon , S. Jo , B. R. Brooks , J. Lee , E. B. Tadmor , H. Heinz , W. Im , J. Chem. Theor. Comput. 2022, 18, 479. 10.1021/acs.jctc.1c00996 PMC8752518 34871001

[185] J. Lee , X. Cheng , J. M. Swails , M. S. Yeom , P. K. Eastman , J. A. Lemkul , S. Wei , J. Buckner , J. C. Jeong , Y. Qi , S. Jo , V. S. Pande , D. A. Case , C. L. Brooks , A. D. MacKerell , J. B. Klauda , J. Chem. Theor. Comput. 2016, 12, 405. 10.1021/acs.jctc.5b00935 PMC4712441 26631602

[186] E. L. Wu , X. Cheng , S. Jo , H. Rui , K. C. Song , E. M. Dávila‐Contreras , Y. Qi , J. Lee , V. Monje‐Galvan , R. M. Venable , J. B. Klauda , W. Im , J. Comput. Chem. 2014, 35, 1997. 25130509 10.1002/jcc.23702 PMC4165794

[187] D. Van Der Spoel , E. Lindahl , B. Hess , G. Groenhof , A. E. Mark , H. J. C. Berendsen , J. Comput. Chem. 2005, 26, 1701. 16211538 10.1002/jcc.20291

[188] N. Kawanishi , H. K. Christenson , B. W. Ninham , J. Phys. Chem. 1990, 94, 4611.

[189] X. Gong , A. Kozbial , L. Li , Chem. Sci. 2015, 6, 3478. 28706709 10.1039/c5sc00832h PMC5492871

[190] K. Vanommeslaeghe , E. P. Raman , A. D. MacKerell Jr , J. Chem. Info. Model. 2012, 52, 3155. 10.1021/ci3003649 PMC3528813 23145473

[191] K. Vanommeslaeghe , E. Hatcher , C. Acharya , S. Kundu , S. Zhong , J. Shim , E. Darian , O. Guvench , P. Lopes , I. Vorobyov , A. D. Mackerell Jr , J Comput. Chem. 2010, 31, 671. 19575467 10.1002/jcc.21367 PMC2888302

[192] K. Vanommeslaeghe , A. D. MacKerell Jr , J. Chem. Inf. Model. 2012, 52, 3144. 23146088 10.1021/ci300363c PMC3528824

[193] D. Horinek , S. I. Mamatkulov , R. R. Netz , J. Chem. Phys. 2009, 130, 124507. 19334851 10.1063/1.3081142

[194] S. Storm , S. Jakobtorweihen , I. Smirnova , A. Z. Panagiotopoulos , Langmuir 2013, 29, 11582. 23941607 10.1021/la402415b

[195] J. B. Klauda , R. M. Venable , J. A. Freites , J. W. O’Connor , D. J. Tobias , C. Mondragon‐Ramirez , I. Vorobyov , A. D. R. W. MacKerellPastor Jr , R. W. Pastor , J. Phys. Chem. B 2010, 114, 7830. 20496934 10.1021/jp101759q PMC2922408

[196] H. Heinz , T.‐J. Lin , R. Kishore Mishra , F. S. Emami , Langmuir 2013, 29, 1754. 23276161 10.1021/la3038846

[197] R. Guixà‐González , I. Rodriguez‐Espigares , J. M. Ramírez‐Anguita , P. Carrió‐Gaspar , H. Martinez‐Seara , T. Giorgino , J. Selent , Bioinformatics 2014, 30, 1478. 24451625 10.1093/bioinformatics/btu037

[198] W. Humphrey , A. Dalke , K. Schulten , J. Mol. Graph. 1996, 14, 33. 8744570 10.1016/0263-7855(96)00018-5

[199] S. Buchoux , Bioinformatics 2017, 33, 133. 27578804 10.1093/bioinformatics/btw563

[200] D. Nečas , P. Klapetek , Open Phys. 2012, 10, 181.

[201] G. Chong , E. D. Laudadio , M. Wu , C. J. Murphy , R. J. Hamers , R. Hernandez , J. Phys. Chem. C 2018, 122, 28393.

[202] X. Wei , A. Popov , R. Hernandez , ACS Appl. Mater. Interfaces 2022, 14, 12538. 35230798 10.1021/acsami.1c24526

[203] R. Savin , N.‐O. Benzaamia , C. Njel , S. Pronkin , C. Blanck , M. Schmutz , F. Boulmedais , Mater. Adv. 2022, 3, 2222.

[204] V. P. Arkhipov , R. V. Arkhipov , N. A. Kuzina , A. Filippov , Magn. Reson. Chem. 2021, 59, 1126. 33864285 10.1002/mrc.5165

[205] H. Hinterwirth , S. Kappel , T. Waitz , T. Prohaska , W. Lindner , M. Lämmerhofer , ACS Nano 2013, 7, 1129. 23331002 10.1021/nn306024a PMC3584655

[206] S. K. Meena , S. Celiksoy , P. Schäfer , A. Henkel , C. Sönnichsen , M. Sulpizi , Phys. Chem. Chem. Phys. 2016, 18, 13246. 27118188 10.1039/c6cp01076h PMC5159743

