# Supplementary material for: Gold Nanoparticle Adsorption and Uptake are Directed by Particle Capping Agent
Source: Small Sci. 2025 May 19;5(7):2500060. doi: 10.1002/smsc.202500060 (PMC12257907; doi:10.1002/smsc.202500060)
Supplement: Supplementary file 1 — Supplementary Material [file SMSC-5-2500060-s001.pdf]

Supporting Information for

Gold Nanoparticle Adsorption and Uptake is Directed by Particle Capping Agent

Rashad Kariuki,[1] Rowan Penman,[1] Alexander D. Newbold,[2] Kalpani A. Mirihana,[1] Pierre H. A. Vaillant,[1] Tilly P. Shepherd,[1] Nastaran Meftahi,[3] Gary Bryant,[1] Kislon Voïtchovsky,[2] Claudia Contini,[4] Andrew Hung,[1] Kevion K. Darmawan,[1] Charlotte E. Conn,[1] Saffron J. Bryant,[1] Andrew J. Christofferson,[1]* and Aaron Elbourne [1,]**

[1]School of Science, STEM College, RMIT University, Melbourne VIC 3001, Australia.
[2]University of Durham, Physics Department, Durham DH1 3LE, UK.
[3]Department of Civil and Construction Engineering, Swinburne University of Technology, Melbourne, Victoria, Australia
[4] Imperial college London, London SW7 2BX, United Kingdom.

* Corresponding Authors
E-mail: andrew.christofferson@rmit.edu.au
E-mail: aaron.elbourne@rmit.edu.au

Supporting Information

Imaging Force Calculation:

The maximum average imaging force (F_{avg}) used during the AM-AFM experiments can be approximated using the following equations; [1]

$$F_{avg} = k_c A_0 \frac{4\pi^2}{3}\left(\frac{\tau}{T}\right)^2 \quad (1)$$

and

$$\frac{\tau}{T} = \frac{arccos\left[\frac{(A_0 - \Delta A)}{A_0}\right]}{2\pi} \quad (2)$$

where, A_0 is the free liquid amplitude (~ 6 nm), ΔA is the change in amplitude upon surface engagement, also referred to as the damped oscillation (~3 – 4 nm), K_c is the cantilever spring constant, T is the period of oscillation of the cantilever, and τ is the contact time of interaction between the tip and the surface. Under these conditions, the max F_{avg} was consistently maintained between ~0.1 nN and 0.2 nN. This value is significantly below the required force to rupture the lipid bilayer (See Figure 2).

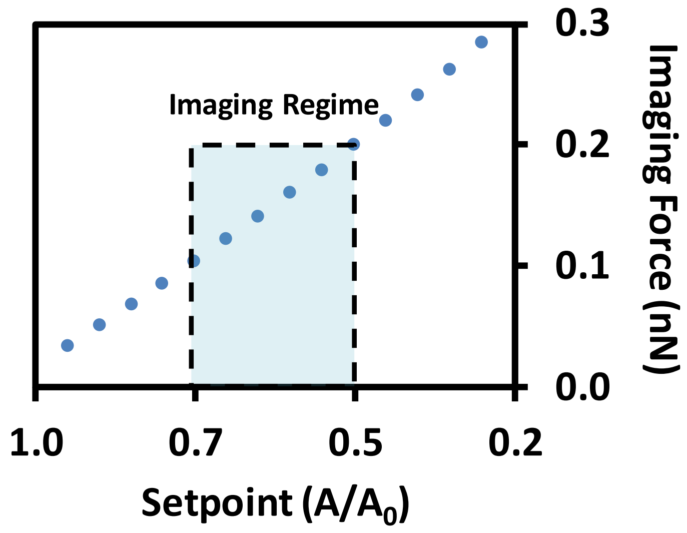

Figure S1. Calculated imaging force as a function of A/A_0. The A/A_0 range used in the experiments here is marked on the graph.

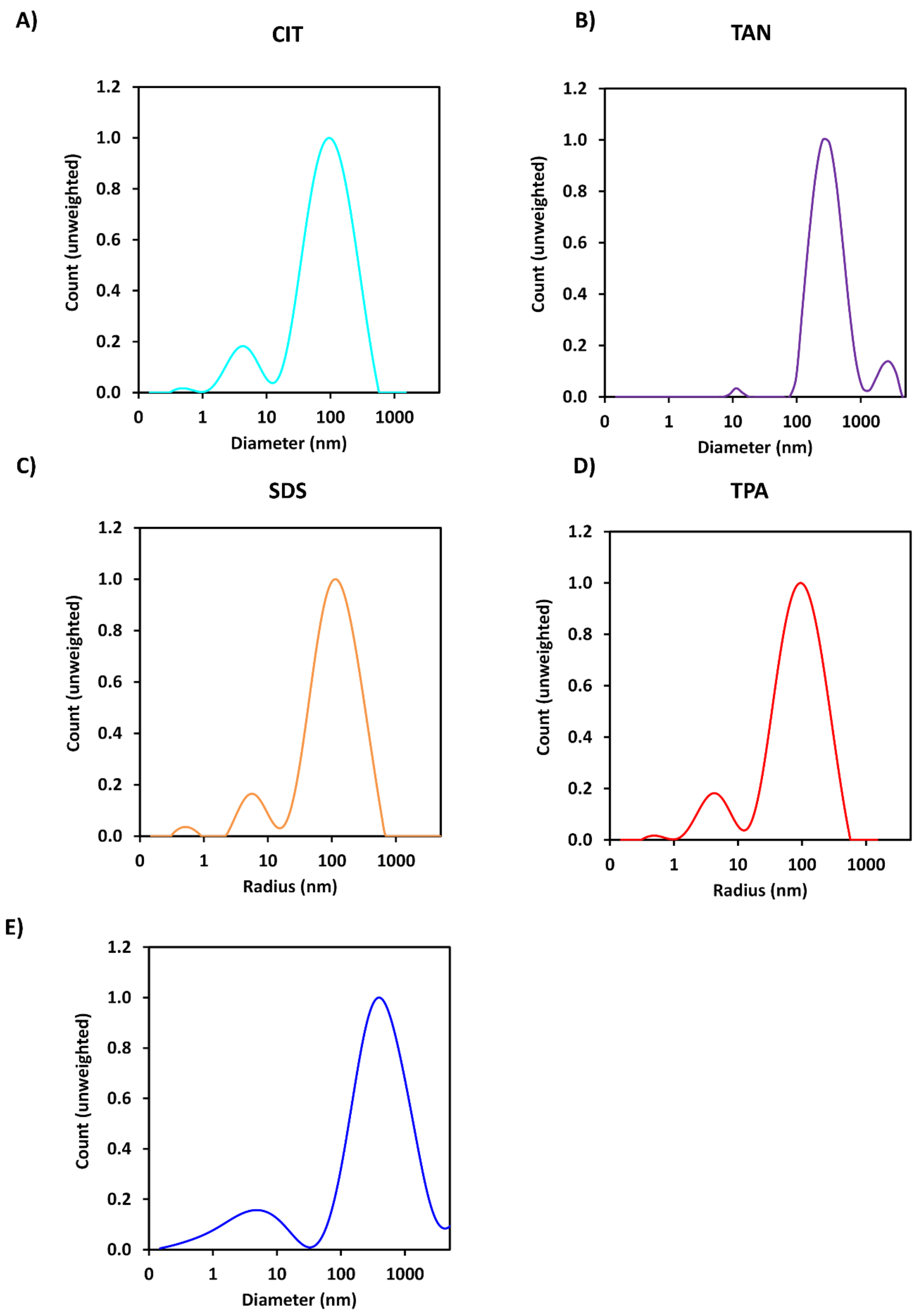

Figure S2. DLS AuNP radial size distribution for (A) CIT (B) TAN (C) SDS (D) TPA (E) CTAB. (Particle concentration was 5×10^{13} particles/mL).

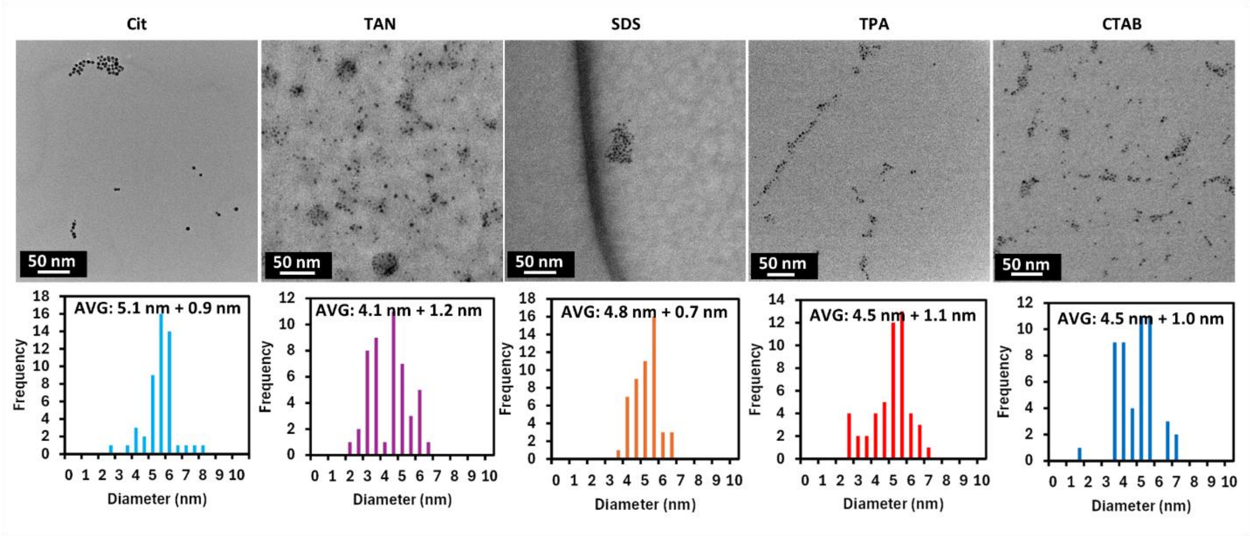

Figure S3. Transmission electron micrograph (TEM) of ligand AuNP systems and size distributions.

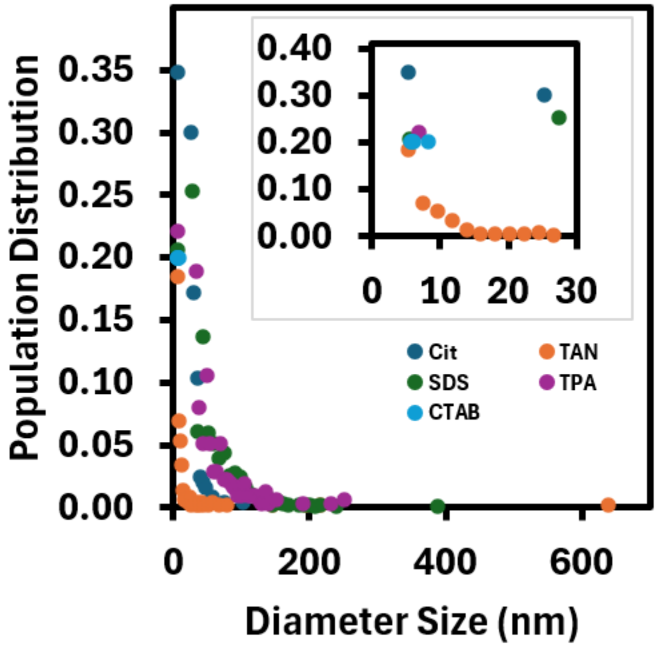

Figure S4. Cluster analysis from the AFM images.

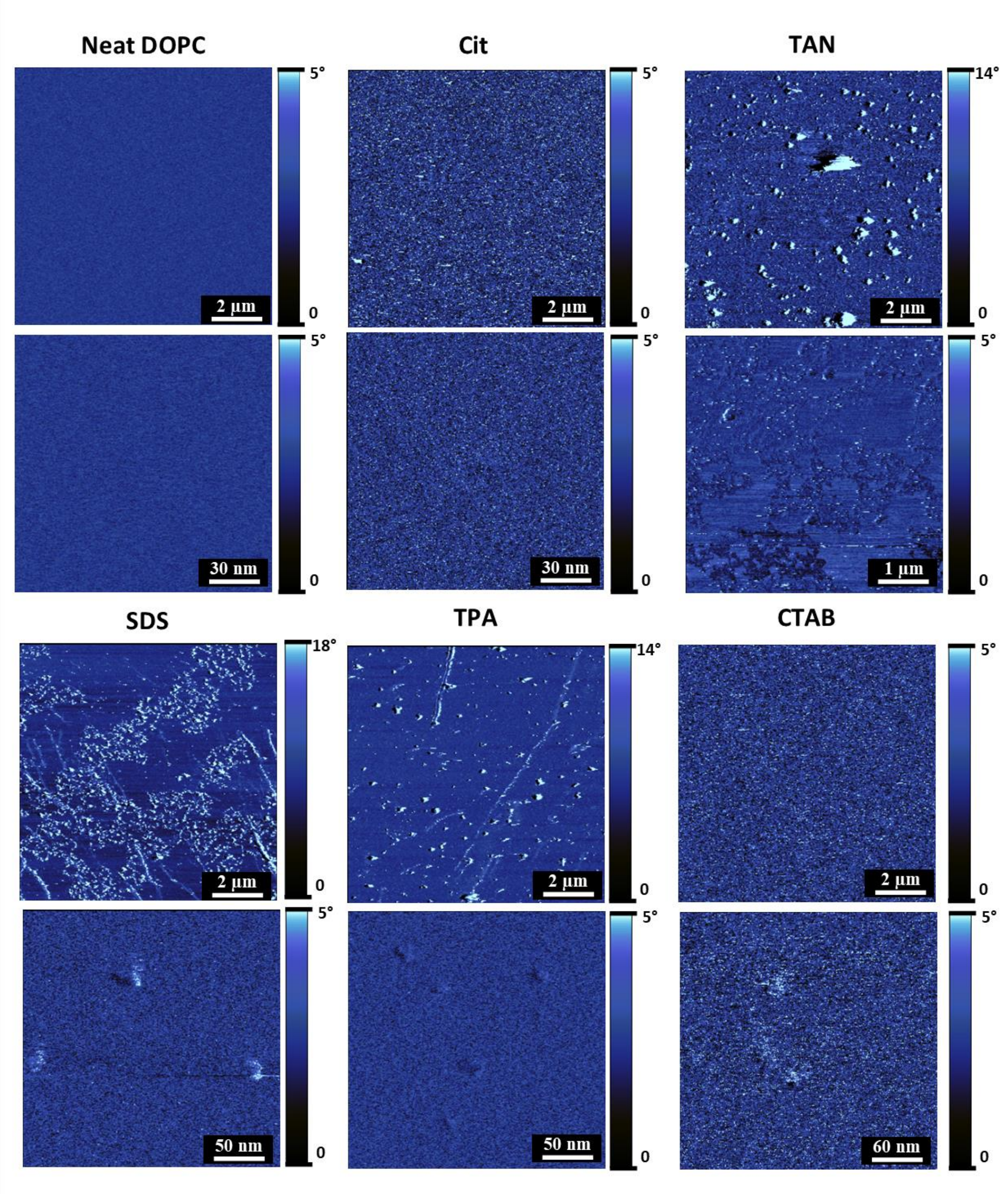

Figure S5. Phase images of the DOPC SLBs obtained following introduction of the AuNPs.

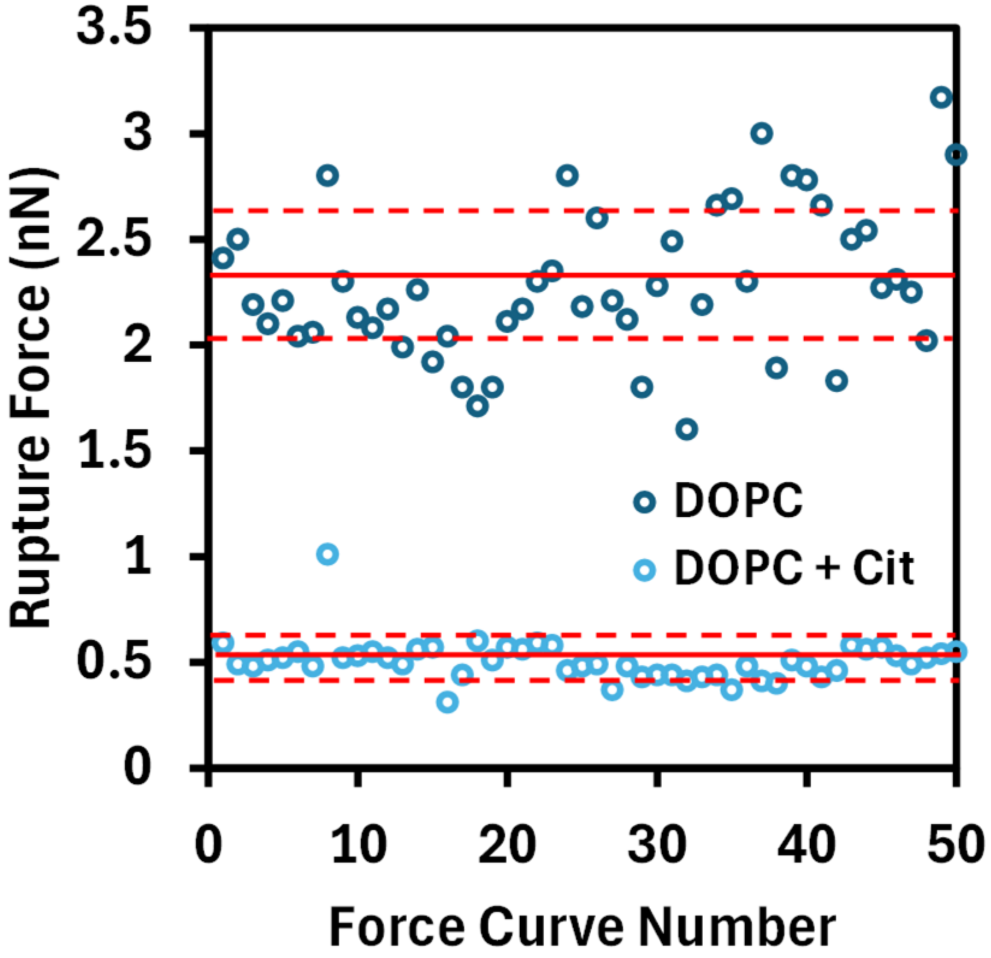

Figure S6. Rupture force evolution profile of DOPC-SLB. In this force spectroscopy experiment, we measured the rupture force of a lipid bilayer using a sharp cantilever tip. To address the concern of potential tip contamination or blunting, we conducted 50 consecutive force measurements and observed that the rupture force remained consistent, demonstraings that contamination is not occurring. If the tip were becoming contaminated with lipid residues or other materials, the rupture force would progressively increase due to changes in the tip's geometry or surface chemistry, resulting in a larger contact area and stronger interactions with the bilayer. Similarly, if the tip were becoming blunt, it would interact with a broader area of the bilayer, altering the force profile and increasing the measured rupture force. The absence of these changes indicates that the tip remains sharp and clean throughout the measurements, ensuring reliable and reproducible results.

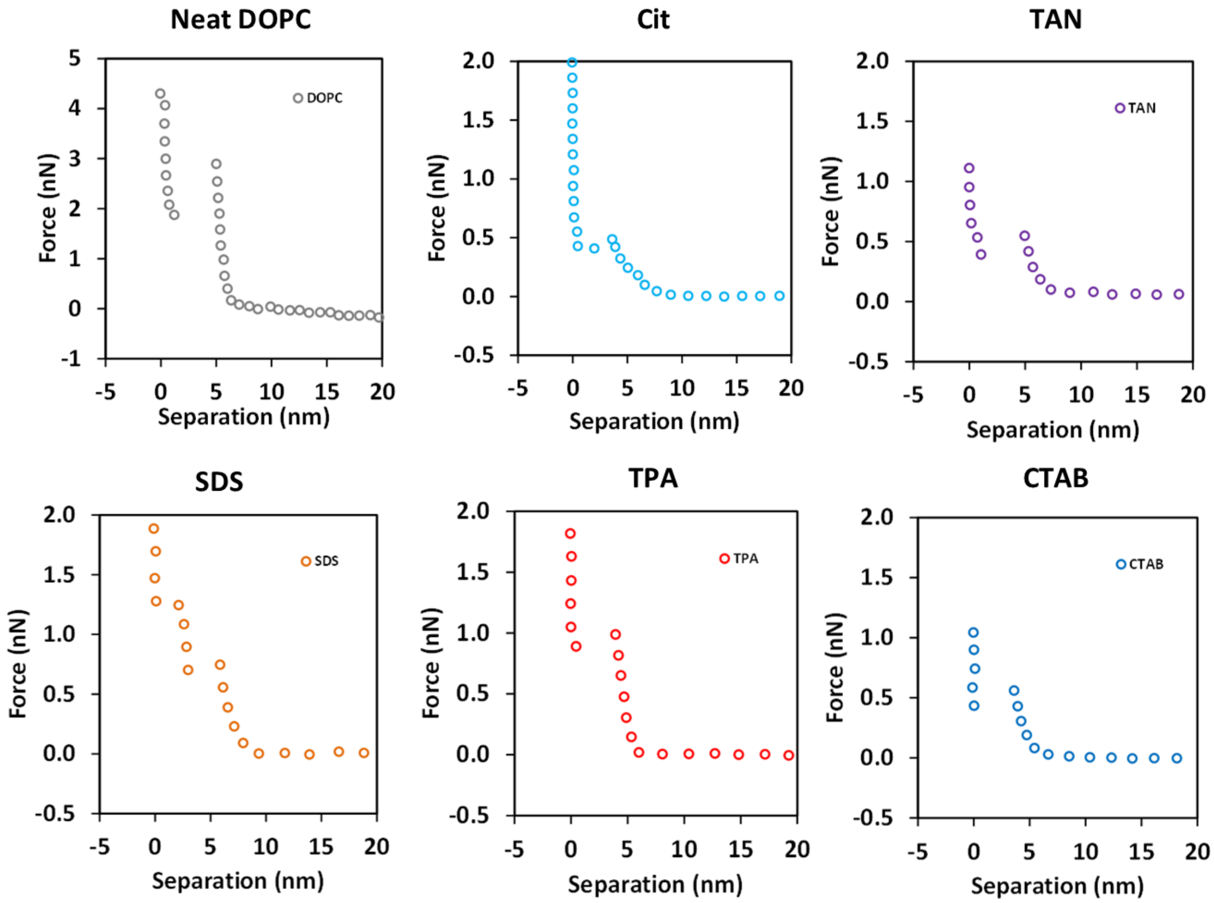

Figure S7. Representative AFM force spectroscopy curves of DOPC-SLB-AuNP-ligand systems.

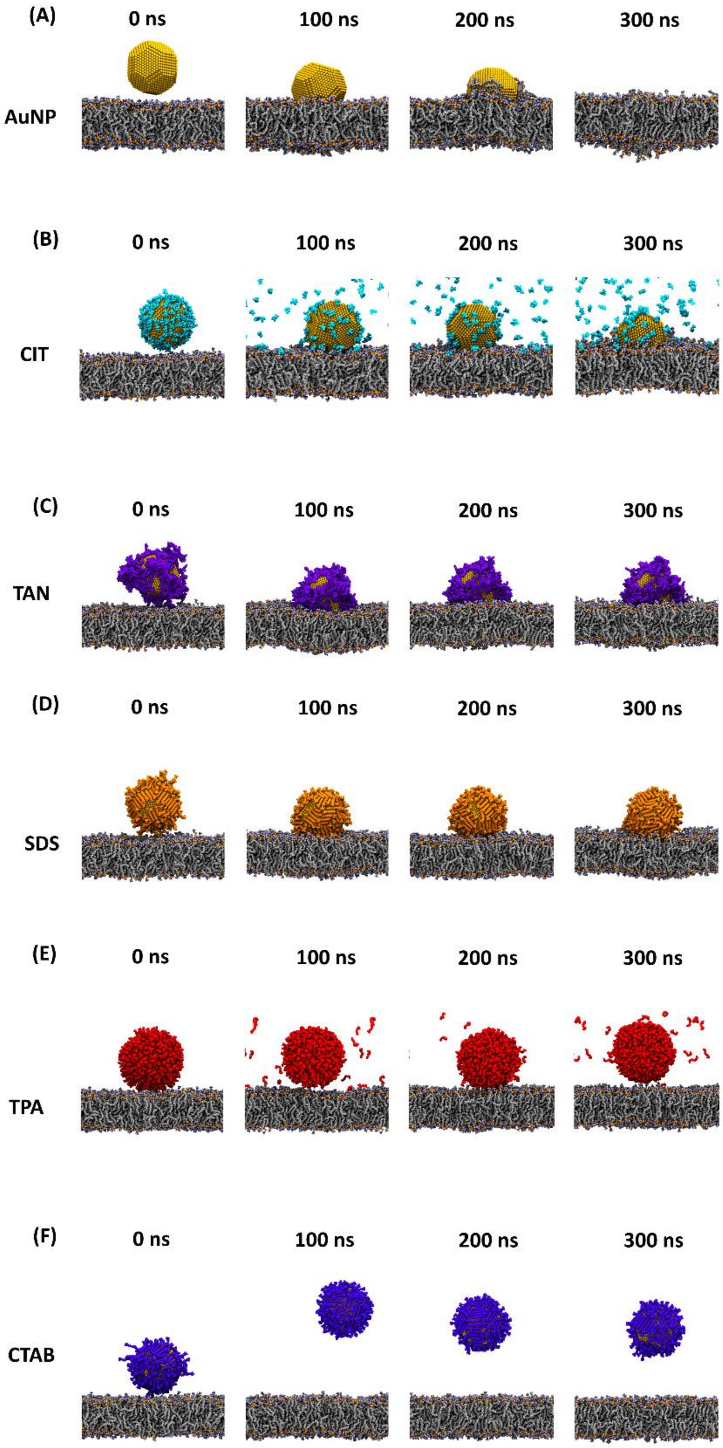

Figure S8. MD simulations of capped AuNP-DOPC (unsupported systems) systems. (A) Citrate (CIT) (B) Tannic acid (TAN) (C) sodium dodecyl sulphate (SDS) (D) Thiol-3-PEG Acid (TPA) (E) Cetyltrimethylammonium bromide (CTAB).

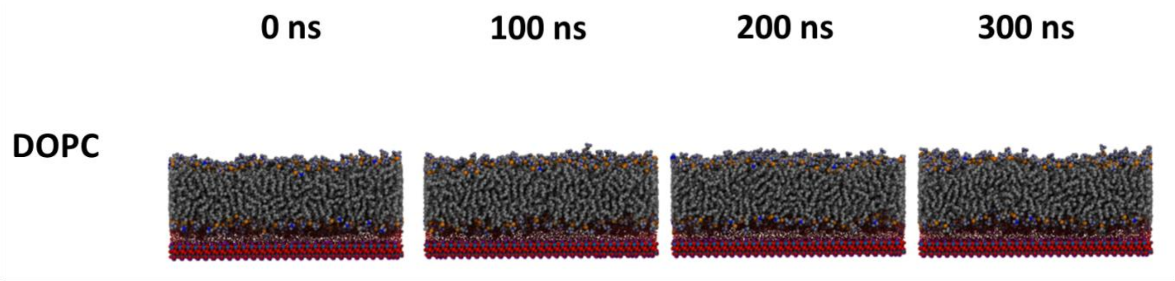

Figure S9. MD simulations of DOPC-SLB

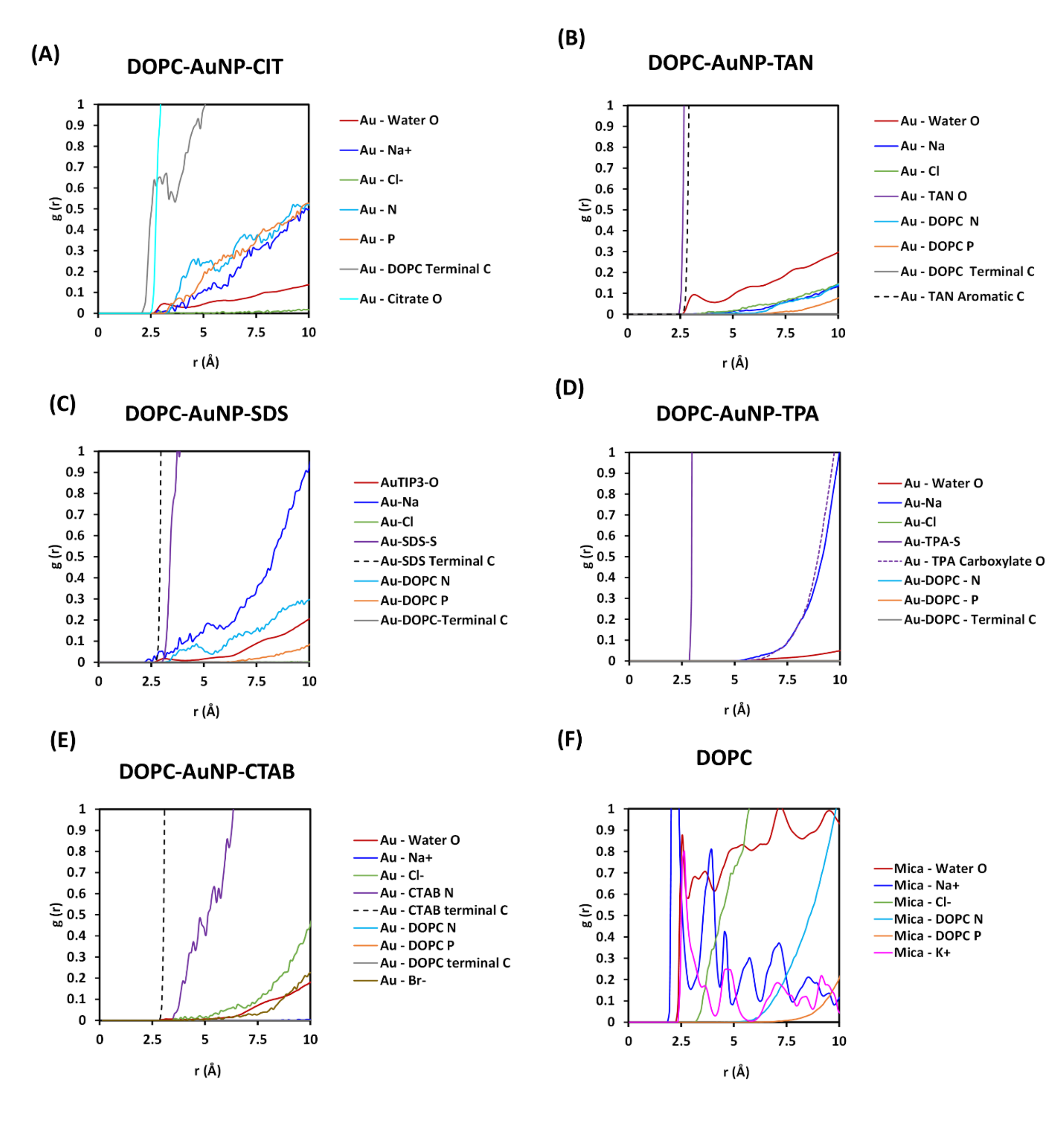

Figure S10. Radial distribution function (RDF) for (A) DOPC-AuNP-SDS. (B) DOPC-AuNP-TPA. (C) DOPC-AuNP-CTAB. (D) DOPC-AuNP-CIT. (E) DOPC-AuNP-TAN. (F) DOPC.

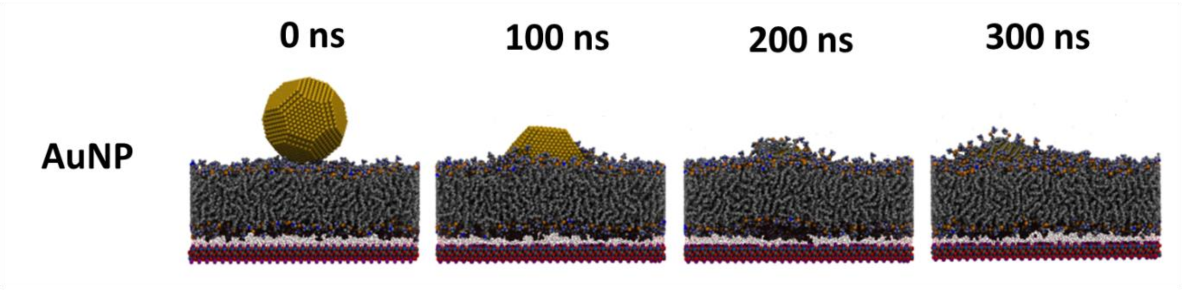

Figure S11. MD simulations of DOPC-SLB with bare AuNP.

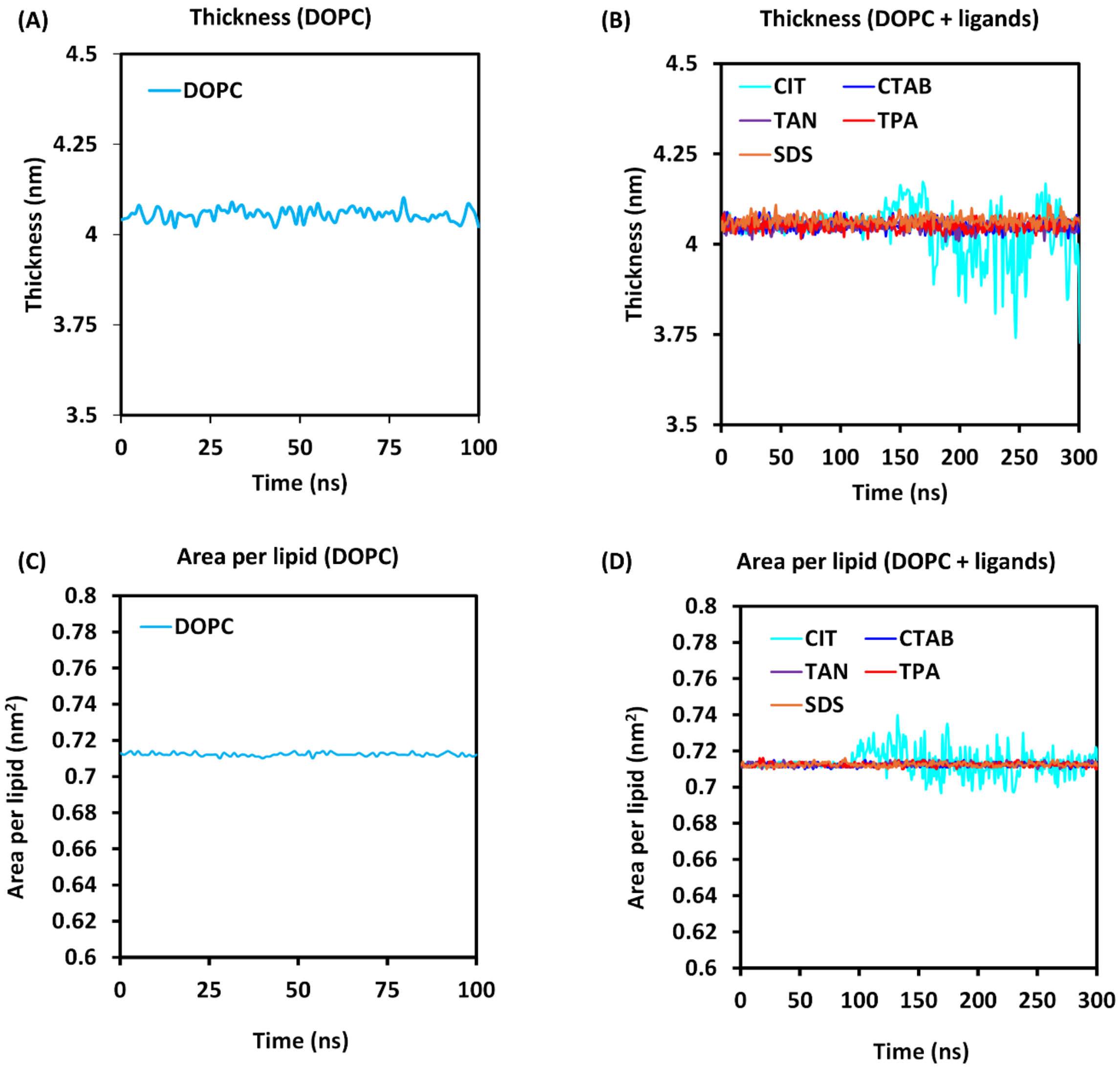

Figure S12. (A) thickness measurement of DOPC and (B) thickness measurements of the DOPC-AuNP-ligand systems. (C) Area per lipid (APL) measurement of DOPC and (D) APL measurements of the DOPC-AuNP-ligand systems

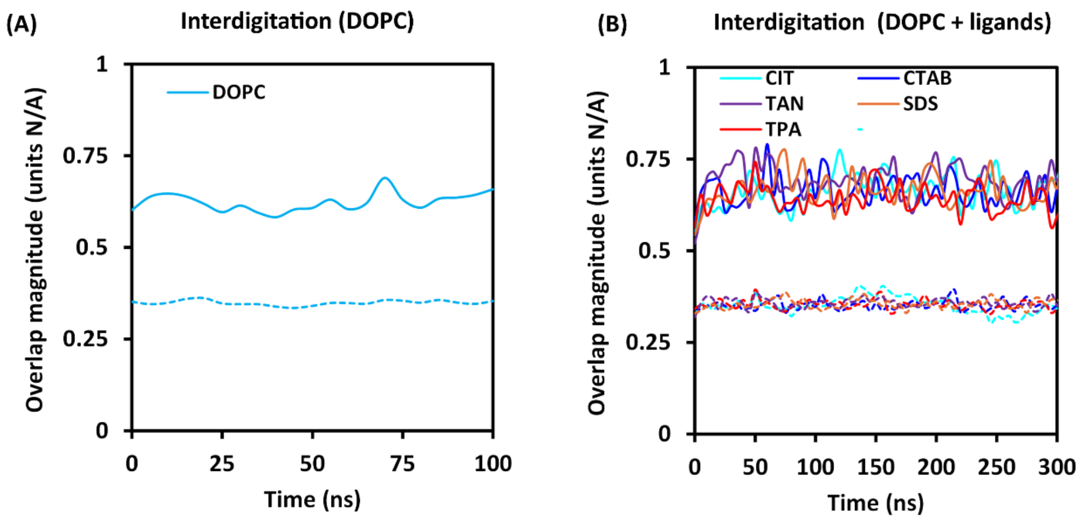

Figure S13. Interleaflet interdigitation as a function of chain carbon chain coupling. Solid line represents raw width in (nm). Dotted line represents fraction of % overlap. (A) DOPC-SLB system. (B) DOPC-AuNP-ligand systems.

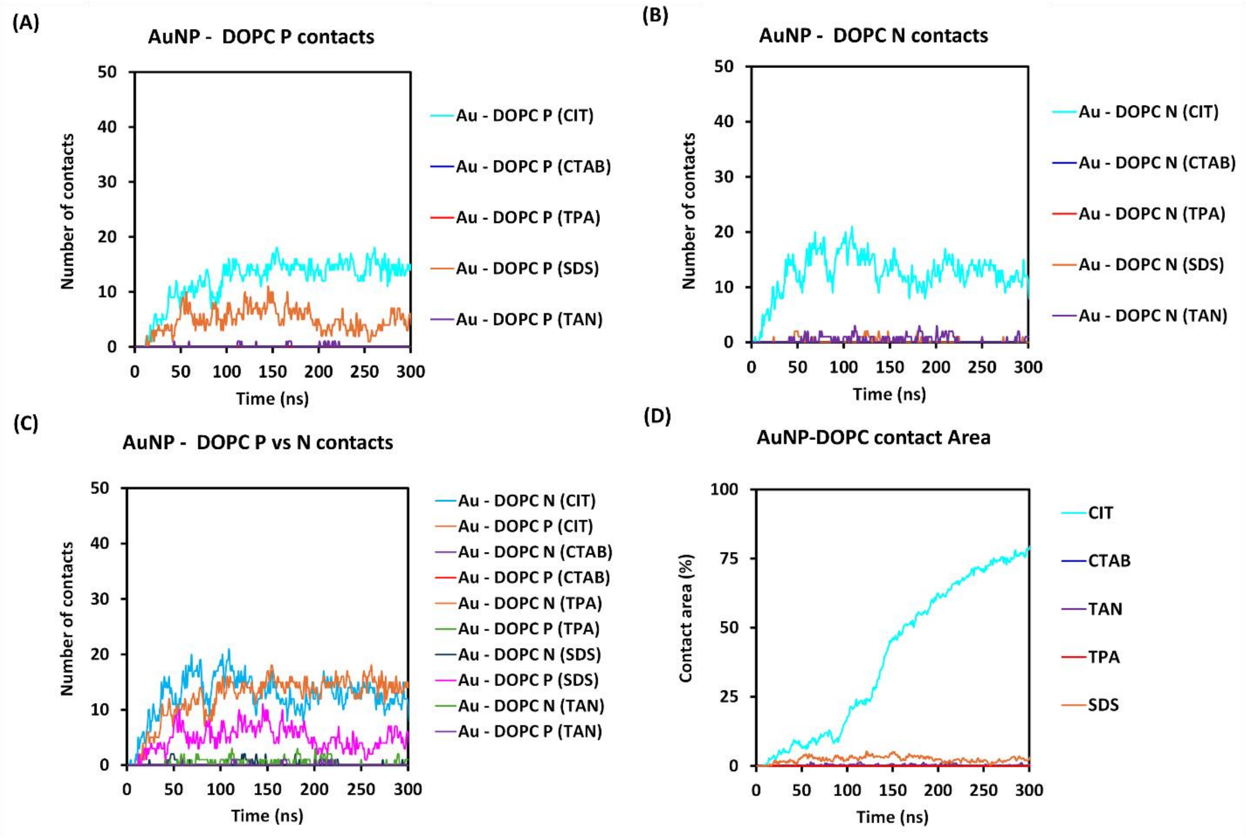

Figure S14. (A) AuNP–DOPC P headgroup contacts as a function of time. (B) AuNP–DOPC N headgroup contacts as a function of time. (C) AuNP–DOPC N vs P headgroup contacts as a function of time. (D) Contact area between the AuNP and the DOPC interface.

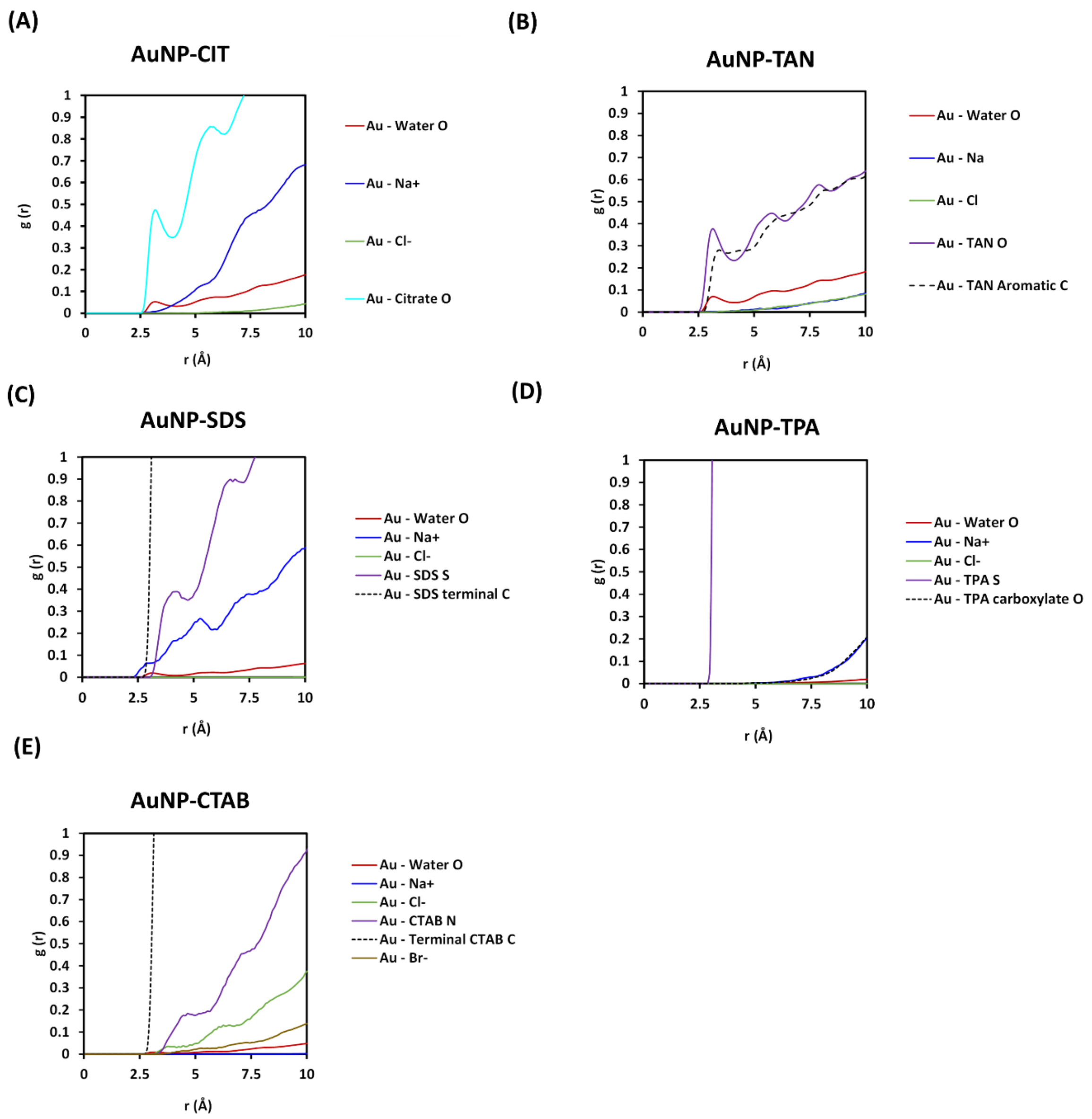

Figure S15. Radial distribution function (RDF) between Au and other species for (A) AuNP-SDS. (B) AuNP-TPA (C) AuNP-CTAB. (D) AuNP-CIT (E) AuNP-TAN.

Table S1. Modified non-bond parameters for pairwise interaction between Au atoms and all other atom types, formatted for the [nonbond_params] section of the GROMACS forcefield.itp file.

i	j	func	sigma	epsilon
AU	HAL1	1	2.49050737E-01	5.67598450E-01
AU	HAL2	1	2.50832534E-01	6.40337411E-01
AU	HAL3	1	2.50832534E-01	5.92837246E-01
AU	HEL1	1	2.42814446E-01	6.73768506E-01
AU	HL	1	1.93815016E-01	8.20746002E-01
AU	HT	1	1.51452782E-01	8.20746002E-01
AU	CEL1	1	3.17649938E-01	9.97893782E-01
AU	CL	1	3.09631849E-01	1.01246234E+00
AU	CTL1	1	3.34131564E-01	5.41183887E-01
AU	CTL2	1	3.10522748E-01	9.05573851E-01
AU	CTL3	1	3.13195444E-01	1.06875254E+00
AU	CTL5	1	3.14977242E-01	1.08236777E+00
AU	NTL	1	2.96268369E-01	1.71137372E+00
AU	O2L	1	2.82904888E-01	1.32562438E+00
AU	OBL	1	2.82904888E-01	1.32562438E+00
AU	OSL	1	2.78450394E-01	1.21012396E+00
AU	OSLP	1	2.78450394E-01	1.21012396E+00
AU	OT	1	2.88980817E-01	1.49243171E+00
AU	SOD	1	2.57135643E-01	8.28736146E-01
AU	PL	1	3.22995330E-01	2.92689938E+00
AU	CLA	1	3.33686115E-01	1.48209311E+00
AU	CG321	1	3.10522748E-01	9.05573851E-01
AU	CG2O2	1	2.82904888E-01	1.19796160E+00
AU	OG2D1	1	2.82904888E-01	1.32562438E+00
AU	CG301	1	3.09631849E-01	6.84549487E-01
AU	OG311	1	2.88695730E-01	1.67723356E+00
AU	HGA2	1	2.50832534E-01	7.15918990E-01
AU	CG2O3	1	3.09631849E-01	1.01246234E+00
AU	HGP1	1	1.51452782E-01	8.20746002E-01
AU	OG2D2	1	2.82904888E-01	1.32562438E+00
AU	IHOY	1	1.79783361E-01	4.68678995E-01
AU	IOY1	1	2.87359382E-01	6.05061980E-01
AU	IOY2	1	2.87359382E-01	6.05061980E-01
AU	IOY3	1	2.87359382E-01	6.05061980E-01
AU	IOY4	1	2.87359382E-01	6.05061980E-01
AU	IOY5	1	2.87359382E-01	6.05061980E-01
AU	IOY6	1	2.87359382E-01	6.05061980E-01
AU	IOY7	1	2.87359382E-01	6.05061980E-01
AU	IOY8	1	2.87359382E-01	6.05061980E-01
AU	IOY9	1	2.87359382E-01	6.05061980E-01
AU	IAY1	1	3.18540837E-01	8.55686859E-01
AU	IAY2	1	3.18540837E-01	8.55686859E-01
AU	IAYT2	1	3.18540837E-01	8.55686859E-01
AU	ISY1	1	3.09631849E-01	8.55686859E-01
AU	ISY2	1	3.09631849E-01	8.55686859E-01
AU	IK_CM	1	3.00722862E-01	1.71137372E+00
AU	SG311	1	3.09631849E-01	6.45543881E+00
AU	OG301	1	2.78450394E-01	1.21012396E+00
AU	HGP3	1	1.71542548E-01	1.21012396E+00
AU	NG3P0	1	2.96268369E-01	1.71137372E+00
AU	CG334	1	3.28786172E-01	1.06187947E+00

AU	HGP5	1	1.93815016E-01	8.20746002E-01
AU	CG324	1	3.25222577E-01	8.97451949E-01
AU	CG331	1	3.14086343E-01	1.06875254E+00
AU	HGA3	1	2.50832534E-01	5.92837246E-01
AU	BRO	1	2.52202106E-01	8.57321410E-01
AU	SL	1	3.18540837E-01	2.62348623E+00
AU	CG2R61	1	3.08954766E-01	1.01246234E+00
AU	HGR61	1	2.52453970E-01	6.62812191E-01
AU	OG302	1	2.78450394E-01	1.21012396E+00
AU	CG311	1	3.09631849E-01	6.84549487E-01
AU	OG3C61	1	2.78450394E-01	1.21012396E+00
AU	HGA1	1	2.50832534E-01	8.11775831E-01

Table S2. AuNP ligand surface behaviour.

**Capping agent**	**Packing type**	**Primary stabilization**	**Structural description**	**References**
**CIT**	loose monolayer	vdW, electrostatics	Dynamic-Solvent exchangeable	[2-7].
**TAN**	Globular network	π-π stacking, hydrophobic interactions, steric stabilization.	Aromatic-Au surface interactions create monolayer tannic acid network with minor protruding side chains.	[8, 9].
**SDS**	Self-assembled monolayer, hydrophobic surface adsorbed tails, solvent facing headgroups	vdW, electrostatics.	Elongated carbon tail on Au-surface with headgroup in solution	[10].
**TPA**	Dense monolayer with loose chain packing (brush-like)	S-Au strong interaction, steric stabilization.	S-Au surface adsorbed monolayer with (allows for tighter packing) and outward facing hydrophilic chain	[11-16]
**CTAB**	Self-assembled monolayer, hydrophobic surface adsorbed tails, solvent facing headgroups	vdW, electrostatics.	Elongated carbon tail on Au-surface with headgroup in solution	[17].

Table S3. Molecular dynamics parameters for the production run.

define	= -DPOSRES -DPOSRES_FC_BB=100.0 -DPOSRES_FC_LIPID=0.0 *
integrator	= md
dt	= 0.002
nsteps	= 15000000
nstxtcout	= 5000
nstvout	= 0
nstfout	= 0
nstcalcenergy	= 100
nstenergy	= 1000
nstlog	= 1000
;	
cutoff-scheme	= Verlet
nstlist	= 20
rlist	= 1.2
coulombtype	= pme
rcoulomb	= 1.2
vdwtype	= Cut-off
vdw-modifier	= Force-switch
rvdw_switch	= 1.0
rvdw	= 1.2
;	;
tcoupl	= Nose-Hoover
tc_grps	= SYSTEM
tau_t	= 1.0
ref_t	= 303.15
;	
constraints	= h-bonds
constraint_algorithm	= LINCS
continuation	= yes
;	;
nstcomm	= 100
comm_mode	= linear

comm_grps		= SYSTEM
;		
refcoord_scaling		= com
periodic-molecules		= yes
; Wall		
nwall	= 2	= 2
wall-atomtype	= WA WA	= WA WA
wall-type	= 12-6	= 12-6
wall-ewald-zfac	= 2	= 2
ewald-geometry	= 3dc	= 3dc
pbc		= xy
wall-r-linpot		= 0.1

*Where = DPOSRES_FC_BB are in reference to the mica atomic restraints (except for K+) and DPOSRES_FC_LIPID are the lipid restraints.

References:

1. Putman, C.A.J., et al., *Tapping mode atomic force microscopy in liquid.* Applied Physics Letters, 1994. **64**(18): p. 2454-2456.
2. Rani, M., et al., *Understanding the mechanism of replacement of citrate from the surface of gold nanoparticles by amino acids: a theoretical and experimental investigation and their biological application.* RSC Advances, 2016. **6**(21): p. 17373-17383.
3. Perera, G.S., et al., *Facile displacement of citrate residues from gold nanoparticle surfaces.* Journal of Colloid and Interface Science, 2018. **511**: p. 335-343.
4. Wei, H., et al., *Real-Time Monitoring of Ligand Exchange Kinetics on Gold Nanoparticle Surfaces Enabled by Hot Spot-Normalized Surface-Enhanced Raman Scattering.* Environmental Science & Technology, 2019. **53**(2): p. 575-585.
5. Cardellini, J., et al., *Membrane Phase Drives the Assembly of Gold Nanoparticles on Biomimetic Lipid Bilayers.* The Journal of Physical Chemistry C, 2022. **126**(9): p. 4483-4494.
6. Montis, C., et al., *Shedding light on membrane-templated clustering of gold nanoparticles.* Journal of Colloid and Interface Science, 2020. **573**: p. 204-214.
7. Wang, X., et al., *Nanoparticle Ligand Exchange and Its Effects at the Nanoparticle–Cell Membrane Interface.* Nano Letters, 2019. **19**(1): p. 8-18.
8. Orlowski, P., et al., *Tannic Acid-Modified Silver and Gold Nanoparticles as Novel Stimulators of Dendritic Cells Activation.* Front Immunol, 2018. **9**: p. 1115.
9. Palafox-Hernandez, J.P., et al., *Optical Actuation of Inorganic/Organic Interfaces: Comparing Peptide-Azobenzene Ligand Reconfiguration on Gold and Silver Nanoparticles.* ACS Applied Materials & Interfaces, 2016. **8**(1): p. 1050-1060.
10. Soares, D.M., W.E. Gomes, and M.A. Tenan, *Sodium Dodecyl Sulfate Adsorbed Monolayers on Gold Electrodes.* Langmuir, 2007. **23**(8): p. 4383-4388.
11. Corbierre, M.K. and R.B. Lennox, *Preparation of Thiol-Capped Gold Nanoparticles by Chemical Reduction of Soluble Au(I)−Thiolates.* Chemistry of Materials, 2005. **17**(23): p. 5691-5696.
12. Lin, J.-Q., et al., *Simulation Study of Aggregations of Monolayer-Protected Gold Nanoparticles in Solvents.* The Journal of Physical Chemistry C, 2011. **115**(39): p. 18991-18998.
13. Heinz, H., et al., *Accurate Simulation of Surfaces and Interfaces of Face-Centered Cubic Metals Using 12−6 and 9−6 Lennard-Jones Potentials.* The Journal of Physical Chemistry C, 2008. **112**(44): p. 17281-17290.
14. Tielens, F. and E. Santos, *AuS and SH Bond Formation/Breaking during the Formation of Alkanethiol SAMs on Au(111): A Theoretical Study.* The Journal of Physical Chemistry C, 2010. **114**(20): p. 9444-9452.
15. Inkpen, M.S., et al., *Non-chemisorbed gold–sulfur binding prevails in self-assembled monolayers.* Nature Chemistry, 2019. **11**(4): p. 351-358.
16. Wright, L.B., et al., *GolP-CHARMM: First-Principles Based Force Fields for the Interaction of Proteins with Au(111) and Au(100).* Journal of Chemical Theory and Computation, 2013. **9**(3): p. 1616-1630.
17. Kalipillai, P., et al., *Self-assembly of a CTAB surfactant on gold nanoparticles: a united-atom molecular dynamics study.* Physical Chemistry Chemical Physics, 2022. **24**(46): p. 28353-28361.
